# Lipidomic markers of habitual physical activity and risk of type 2 diabetes in American Indians

**DOI:** 10.1038/s41598-025-29211-y

**Published:** 2025-11-24

**Authors:** Xiaoxiao Wen, Guanhong Miao, Mingjing Chen, Ying Zhang, Mary Mohr, Amanda M. Fretts, Oliver Fiehn, Jinying Zhao

**Affiliations:** 1 Health Informatics Institute, University of South Florida, 3650 Spectrum Boulevard, Tampa, FL USA; 2 Department of Neurology at Johns Hopkins University, Baltimore, MD USA; 3 Center for American Indian Health Research, Department of Biostatistics and Epidemiology, University of Oklahoma Health Sciences Center, Oklahoma City, OK USA; 4 MedStar Health Research Institute, Hyattsville, MD USA; 5 Department of Epidemiology, University of Washington, Seattle, WA USA; 6 West Coast Metabolomics Center, University of California-Davis, Davis, CA USA

**Keywords:** Lipidomics, Physical activity, Type 2 diabetes, American indian, Diseases, Endocrinology, Health care, Medical research, Risk factors

## Abstract

Research is needed to identify lipidomic markers associated with habitual physical activity (PA) among American Indian adults and examine their associations with diabetes risk. We performed LC-MS to quantify 1,542 fasting plasma lipids from 1,779 participants at baseline and 1,406 at follow-up (about 5.5-year apart). PA was objectively measured using pedometers at both visits. We identified lipids associated with PA using mixed-effects linear regression, adjusting for BMI and other covariates. Logistic and linear regression was used to evaluate the associations of PA-related lipids with incident diabetes/prediabetes and glucose/insulin metrics. Mediation role of lipids was examined. Among 1,047 participants with normal fasting glucose at baseline, 68 developed diabetes and 178 developed prediabetes. PA was associated with 127 lipids (q < 0.05), including positive associations with glycerophospholipids and inverse associations with triacylglycerols. Eighteen of the 36 annotated lipids were associated with incident diabetes/prediabetes, with 16 linked to lower PA and higher diabetes risk. Most PA-related lipids were also associated with changes in glucose/insulin metrics, and 28 lipids mediated the positive association between PA and insulin sensitivity. Overall, we identified lipidomic signatures of PA among American Indians. These lipids were associated with diabetes risk, potentially through mediating the protective effects of PA on insulin sensitivity.

**Supplementary Information:**

The online version contains supplementary material available at 10.1038/s41598-025-29211-y.

## Introduction

American Indians suffer a disproportionately high burden of type 2 diabetes compared to other racial and ethnic groups^1^. Dyslipidemia, characterized by abnormal lipid levels, including elevated high total cholesterol, high low-density lipoprotein cholesterol (LDL-c), low high-density lipoprotein cholesterol (HDL-c), and high triacylglycerols (TAGs), is a significant risk factor for diabetes and related metabolic disorders such as cardiovascular disease^2,3^. Physical activity, conversely, is a key modifiable lifestyle factor that can reduce the risk of both dyslipidemia and diabetes^4^. Our previous research in the Strong Heart Study of American Indians has shown that higher levels of habitual physical activity are associated with a lower risk of diabetes^5^ and improvements in lipid profiles^6^. The high prevalence of dyslipidemia and the elevated risk of diabetes in this population underscore the urgent need to further investigate the role of physical activity in the development and prevention of these chronic metabolic conditions.

Lipidomics is a high-throughput biochemical technology that enables comprehensive profiling of individual lipid species within biological samples. Previous studies have identified associations of blood metabolites (e.g., branched-chain amino acids [BCAAs] and glutamate) and lipids (e.g., cholesterol esters[CEs], phosphatidylethanolamines [PEs] and plasmalogens) with habitual physical activity^7–12^, as well as cardiometabolic disorders^13–18^ in various populations including American Indians. Despite these advances, several important research gaps remain. First, the lipidome coverage in prior studies has been relatively limited, potentially missing lipid species that are responsive to physical activity. This is particularly crucial, as emerging evidence suggests that altered lipid concentrations represent major metabolomic changes associated with long-term physical activity^12,19^. Second, most existing studies have employed cross-sectional designs, measuring metabolites or lipids associated with response to physical activity at only a single time point, thereby limiting the ability to assess their longitudinal associations. Third, the majority of studies rely on self-reported physical activity data, which are subject to recall bias and social desirability bias, potentially compromising data accuracy.

To address these gaps, we leveraged longitudinal lipidomics data comprising 1,542 individual lipid species from 14 lipid classes measured in 3,907 fasting plasma samples from 1,958 unique American Indian participants at two time points (about 5.5 years apart) in the Strong Heart Family Study (SHFS). In our previous work^18^, we have identified lipidomic markers associated with type 2 diabetes, e.g., multiple glycerophospholipids, triacylglycerols, diacylglycerols, and other lipid species. The current study extended our previous research by investigating the relationship between physical activity, lipidomic remodeling, and risk of diabetes. This study aims to: (a) examine the associations between each individual lipid species and objectively measured physical activity levels at both time points; (b) evaluate whether physical activity-associated lipids are prospectively associated with incident diabetes and prediabetes; (c) assess whether physical activity-associated lipids are longitudinally associated with markers of glucose and insulin homeostasis; and (d) determine whether a lipidomic score of physical activity, derived from the identified lipids, is associated with future diabetes risk. Additionally, we examined the potential mediating role of lipid species in the relationship between physical activity and glucose/insulin homeostasis.

## Methods

### Study population

The SHFS is a multicenter, family-based prospective study designed to identify genetic, metabolic and behavioral factors for cardiovascular disease, diabetes and their risk factors among American Indians. Briefly, a total of 2,780 tribal members (aged ≥ 14 years) residing in Arizona, North Dakota, South Dakota, and Oklahoma were recruited and examined in 2001–2003 and re-examined in 2006–2009. Detailed descriptions of the SHFS protocols for data collection have been described previously^20^. All participants received a personal interview and a physical examination at each visit, during which fasting blood samples were collected for laboratory tests. Laboratory methods were reported previously^21^. For the current analyses, we included participants who: (a) were free of overt cardiovascular disease at baseline, (b) had available data on physical activity, and (c) had available lipidomics data.

All participants provided informed consent at baseline. The SHFS protocols were approved by the Institutional Review Boards of the participating institutions and the American Indian Tribes. All methods were carried out in accordance with the relevant guidelines and regulations of the participating institutions.

### Assessments of habitual physical activity levels

Habitual physical activity was quantified using Accusplit AE120 pedometers at baseline and follow-up. Participants were asked to wear the pedometers for 7 consecutive days (5 weekdays and 2 weekend days) at all times, except while bathing, swimming, or sleeping. We used steps/day as measures of usual physical activity levels, which was calculated by averaging the total number of steps recorded each day during the 7-day period.

### Ascertainment of incident diabetes and prediabetes

Incident type 2 diabetes and prediabetes were ascertained in those who had normal fasting glucose at baseline (2001–2003) but developed diabetes/prediabetes by end of follow-up (2006–2009). Incident diabetes was defined as fasting plasma glucose ≥ 126 mg/dL or receiving hypoglycemic medication according to the American Diabetes Association criteria. Incident prediabetes was defined as fasting plasma glucose of 100–125 mg/dL.

### Measures of glucose and insulin homeostasis

Glucose and insulin homeostasis metrics include fasting plasma glucose, HOMA-IR (i.e., insulin resistance), and the quantitative insulin sensitivity check index (QUICKI). Data on the three metrics were obtained for both baseline and follow-up. Fasting glucose was measured by standard laboratory methods^20^. Insulin resistance was assessed by HOMA-IR = fasting glucose (mg/dL) × insulin (µU/mL)/405^22^. Insulin sensitivity was estimated by QUICKI = 1/(log insulin [mU/L] + log baseline glucose [mg/dL])^23^.

### Assessments of covariates

Sociodemographic information (age, sex and education), alcohol drinking status, smoking status, medical history, and use of prescription medications were collected using structured questionnaires^20,21^. For alcohol drinking, participants were classified as current/former/never drinkers. Smoking status was categorized as current/former/never smokers. Anthropometric measures including height and weight, and fasting blood tests were obtained through physical examinations at each visit. BMI was calculated as body weight in kilograms divided by the square of height in meters. Information on use of anti-hypertensive medication, anti-diabetic medication and lipid-lowering medication was collected at each visit. Hypertension was defined as measured systolic blood pressure ≥ 140 mmHg, diastolic blood pressure ≥ 90 mmHg, or use of antihypertensive medications.

### Lipidomic data acquisition, processing and normalization

Detailed methods for lipidomic data acquisition, processing and normalization in the SHFS are provided in the **Supplemental Methods**. Briefly, relative abundance of molecular lipid species in fasting plasma samples at baseline and follow-up was quantified by untargeted liquid chromatography–mass spectrometry (LC–MS). After preprocessing and quality control, we obtained data of 1,542 lipid species (518 known, 1,024 unknown) across 14 known lipid classes from 1,957 participants at baseline, and 1,948 participants at follow-up.

### Inclusion and exclusion criteria

Among participants with available lipidomics data, we further excluded those with missing information on physical activity or covariates. The final sample included 1,779 participants at baseline and 1,406 at follow-up. **Supplemental Figure ****S1** shows the flowchart of participant selection and statistical analyses, including sample sizes after each exclusion step.

### Statistical analyses

Statistical analyses were performed using R version 4.1.1 (R Foundation for Statistical Computing, Vienna, Austria). We standardized lipidomics data and continuous variables to zero mean and unit variance before the analyses. **Supplemental Figure ****S1** shows the flowchart for participants selection and statistical analyses. The following statistical analyses were performed:

### Lipid species associated with habitual physical activity

To capture the longitudinal associations, we used mixed-effects linear regression to identify lipid species associated with physical activity across visits. The two rounds of lipidomic and physical activity data were treated as repeated measurements for each individual. We used a prediction model approach, treating lipids as potential biomarkers of physical activity. Accordingly, the relative abundance of each lipid was modeled as the independent variable, and physical activity levels (steps/day) as the dependent variable. Covariates were determined based on prior literature, and included age, sex, study center, education (years), BMI (kg/m^2^), fasting glucose (mg/dL), alcohol drinking (never/former/current), smoking (never/former/current), lipid-lowering medication use (yes/no), and hypertension status (yes/no) at the study examination. The family relatedness among the participants and correlation within the same individual between two time points were accounted for by including random intercept terms in these models. Multiple testing was corrected using the Storey’s q-value method^24^, wherein statistical significance was set at a two-sided q < 0.05 level.

### Physical activity-related lipids associated with incident diabetes/prediabetes

We then applied mixed-effects logistic models to estimate the odds ratio (OR) of each physical activity-related lipid for incident diabetes or prediabetes. Incident diabetes, incident prediabetes or combined incident diabetes/prediabetes was the dependent variable separately, and baseline level of each lipid was the independent variable. The same set of covariates described above (at baseline level) was adjusted in the model.

### Physical activity-related lipids associated with glucose/insulin metrics

We used mixed-effects linear regression models to examine the longitudinal associations of physical activity-related lipids and three glucose/insulin metrics, i.e., fasting glucose, HOMA-IR and QUICKI. These glucose/insulin metrics were measured at both baseline and follow-up examinations. In these models, each lipid is the independent variable, each glucose/insulin measure was the dependent variable, and we adjusted for the same set of covariates described above except fasting glucose. Fasting glucose was not included as a covariate in analyses of glucose- or insulin-related outcomes to avoid potential over-adjustment. We accounted for the family relatedness among the participants and correlation within the same individual between two time points by including random intercept terms in these models. Furthermore, we examined the potential mediation roles of the identified lipids in the associations of physical activity levels with the glucose/insulin metrics, using the Mediation R package^25^. Mediation proportion of lipids was calculated as the average causal mediation effects relative to the total effect. Based on 1,000 Monte Carlo draws for quasi-Bayesian approximation, we calculated the 95%CI for the mediation proportion.

### Pathway enrichment analyses

To identify potential metabolic pathways underlying the effects of physical activity, we analyzed the significant lipids (*P* < .05) in the MetaboAnalyst 6.0 platform^26^. We used lipids with nominal *P* < .05 as input, allowing pathway enrichment to detect biologically relevant patterns that might be missed if only qvalue-significant lipids were included. The statistical significance of each pathway was evaluated using the Fisher’s exact test. We controlled for multiple testing in the pathway analysis using a false discovery rate (FDR) threshold of < 0.05. The importance of each pathway was measured by topological analysis^27^. The pathway impact score was calculated by dividing the sum of the importance measures of the identified lipids by the total importance measures of all metabolites within the pathway.

### Lipidomic score of habitual physical activity

First, we constructed a lipidomic score of habitual physical activity using the identified lipids (known) at q < 0.05. Consistent with the approach used in previous studies^28–30^, the lipidomic score was calculated as the sum of standardized lipid concentrations weighted by their corresponding regression coefficients from single lipid models. Then, we investigated whether the lipidomics score at baseline was associated with incident diabetes or prediabetes, and glucose/insulin metrics. We used mixed-effects logistic regression for the analyses of incident diabetes or prediabetes, and mixed-effects linear regression for the analyses of glucose/insulin metrics. All analyses adjusted for the same covariates used in single lipid models.

### Sensitivity analyses

To account for potential confounding by diet, we further adjusted for diet quality in the main analyses. Diet quality was assessed using the Alternative Healthy Eating Index-2010 (AHEI) based on the Block Food Frequency Questionnaire and an American Indian food questionnaire. The dietary data collection and AHEI calculation in SHFS have been described in detail previously^31^.

## Results

### Characteristics of study participants

Table 1 shows the characteristics of the participants at each study examination by physical activity levels. We included 1,779 participants at baseline and 1,406 at follow-up (about 63% female). At baseline, mean age was 40 years and mean BMI was 32 kg/m^2^; about 29% of the study participants had hypertension and 18% had diabetes. On average, participants walked 5,790 and 6,286 steps per day at baseline and follow-up respectively. Those with higher steps per day were more likely to be younger, male, have lower BMI, blood pressure and fasting glucose, and a lower prevalence of hypertension and diabetes at both baseline and follow-up.

**Table 1:** Characteristics of the American Indian participants in the strong heart family study (Baseline 2001–2003, Follow-up 2006–2009) by physical activity levels, united States.

Characteristics	Baseline				Follow-up			
	(Median: 5054 steps)				(Median: 5439 steps)			
	All	< median	≥ median	*P*	All	< median	≥ median	*P*
	(*n* = 1779)	(*n* = 889)	(*n* = 890)		(*n* = 1406)	(*n* = 703)	(*n* = 703)	
Age, years	40.3 (13.9)	43.6 (14.5)	37.1 (12.4)	< 0.001	46.1 (13.7)	48.9 (14.3)	43.4 (12.4)	< 0.001
Female, n (%)	1119 (62.9)	658 (74.0)	461 (51.8)	< 0.001	892 (63.4)	504 (71.7)	388 (55.2)	< 0.001
Education, years	12.6 (2.1)	12.5 (2.2)	12.7 (2.0)	0.065	12.8 (2.1)	12.7 (2.2)	12.9 (2.1)	0.061
BMI, kg/m^2^	31.8 (7.4)	33.0 (8.0)	30.5 (6.6)	< 0.001	32.7 (7.7)	34.2 (8.4)	31.3 (6.7)	< 0.001
Smoking status, n (%)				0.104				0.652
Never	648 (36.4)	319 (35.9)	329 (37.0)		533 (37.9)	268 (38.1)	265 (37.7)	
Former	419 (23.6)	228 (25.6)	191 (21.5)		366 (26.0)	189 (26.9)	177 (25.2)	
Current	712 (40.0)	342 (38.5)	370 (41.6)		507 (36.1)	246 (35.0)	261 (37.1)	
Drinking status, n (%)				< 0.001				< 0.001
Never	144 (8.1)	89 (10.0)	55 (6.2)		150 (10.7)	99 (14.1)	51 (7.3)	
Former	524 (29.5)	293 (33.0)	231 (26.0)		460 (32.7)	254 (36.1)	206 (29.3)	
Current	1111 (62.5)	507 (57.0)	604 (67.9)		796 (56.6)	350 (49.8)	446 (63.4)	
Systolic blood pressure, mmHg	122.4 (15.4)	124.0 (15.7)	120.8 (15.0)	< 0.001	123.0 (16.3)	124.6 (16.7)	121.4 (15.8)	< 0.001
Fasting glucose, mg/dL	109.5 (46.2)	116.3 (53.4)	102.8 (36.5)	< 0.001	113.3 (53.3)	119.2 (58.6)	107.5 (46.8)	< 0.001
Hypertension, n (%)	513 (28.8)	306 (34.4)	207 (23.3)	< 0.001	525 (37.3)	305 (43.4)	220 (31.3)	< 0.001
Diabetes, n (%)	328 (18.4)	222 (25.0)	106 (11.9)	< 0.001	344 (24.5)	220 (31.3)	124 (17.6)	< 0.001
Lipid-lowering medicine use, n (%)	59 (3.3)	34 (3.8)	25 (2.8)	0.288	160 (11.4)	95 (13.5)	65 (9.2)	0.015
Physical activity, steps/day	5790.2 (3866.0)	3054.3 (1263.1)	8523.1 (3651.8)	< 0.001	6285.9 (4754.8)	3162.5 (1423.6)	9409.4 (4866.3)	< 0.001

Continuous variables were expressed as mean (standard deviation) and categorical variables as n (%).

P values were calculated to test differences between < median and ≥ median groups, using t-tests for continuous variables and chi-squared tests for categorical variables.

### Lipid species associated with habitual physical activity

**Fig. 1:**
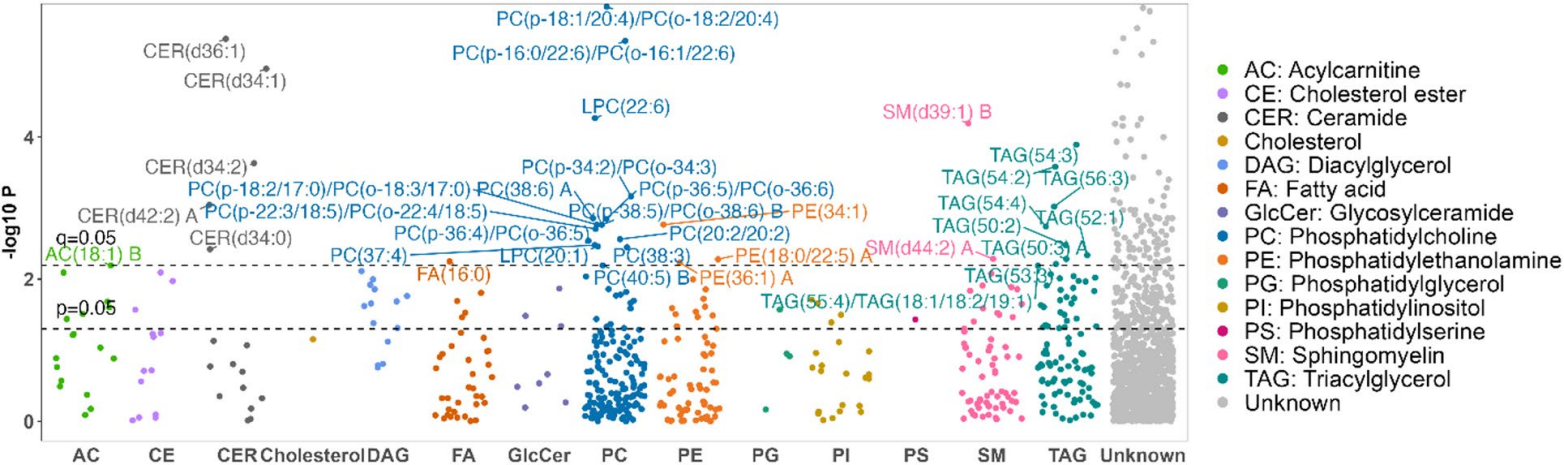
Manhattan plot showing the association of individual lipid species with habitual physical activity. P-values for associations were obtained by mixed-effects linear regression, adjusting for age, sex, study center, education, BMI, fasting glucose, smoking, alcohol use, lipid-lowering medication use, and hypertension status at the study visit. X-axis: lipid classes; Y-axis: -log^10^ P. Different colors represent different lipid classes. The dashed line represents significance level at q = 0.05 or *P* = .05. The letter A or B at the end of lipid names represents isomers.

After multiple testing correction, we identified 127 lipid species (36 known) associated with physical activity levels (q < 0.05), independent of BMI and multiple socio-demographic, lifestyle and clinical covariates (Fig. 1).

**Fig. 2:**
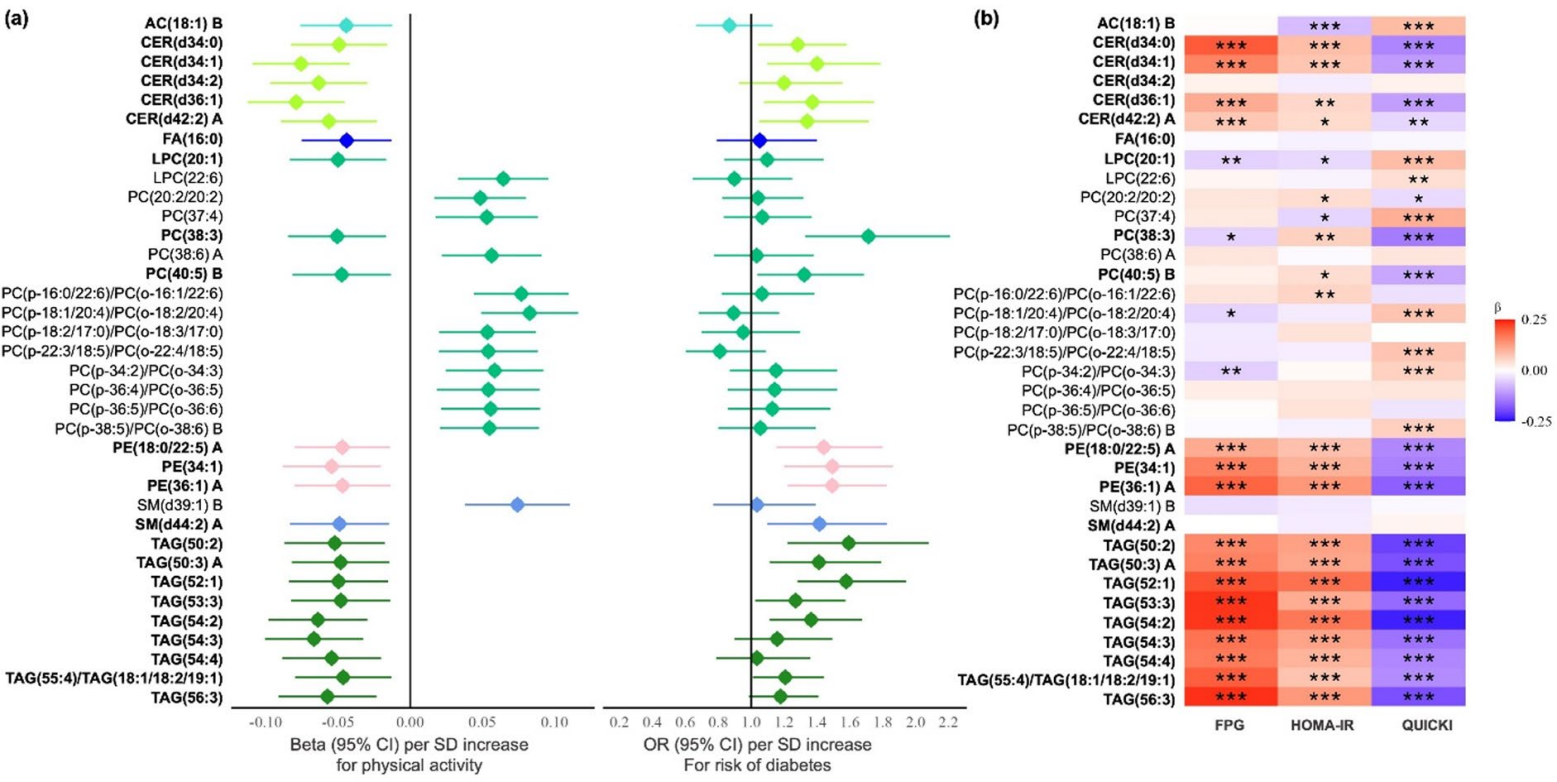
(**a**) Plasma lipid species associated with physical activity levels (q < 0.05) and diabetes risk. Beta coefficients of lipids associated with physical activity were obtained by mixed-effects linear regression models, adjusting for age, sex, study center, education, BMI, fasting glucose, smoking, alcohol use, lipid-lowering medication use, and hypertension status at the study visit. Odds ratios of baseline lipids associated with incident diabetes were obtained by mixed-effects logistic models, adjusting for all covariates above (baseline level). Lipids in boldface have inverse associations with habitual physical activity. The letter A or B at the end of lipid names represents isomers. Abbreviations: AC, acylcarnitine; CER, Ceramide; FA, fatty acid; LPC, lysophosphatidylcholine; OR, odds ratio; PC, phosphatidylcholine; PE, phosphatidylethanolamine; SD, standard deviation; SM, sphingomyelin; TAG, triacylglycerol. (**b**) Associations between physical activity-related lipids (q < 0.05) and glucose/insulin homeostasis metrics (*N* = 1,277). Regression coefficients were obtained from mixed-effects linear regression models, adjusting for age, sex, study center, education, BMI, smoking, alcohol use, lipid-lowering medication use and hypertension. Red color represents positive associations and blue represents inverse associations. Lipids in boldface have inverse associations with habitual physical activity.* *P* < .05, ** *P* < .01, *** *P* < .001. Abbreviations: AC, acylcarnitine; CER, Ceramide; FA, fatty acid; FPG, fasting plasma glucose; LPC, lysophosphatidylcholine; PC, phosphatidylcholine; PE, phosphatidylethanolamine; SM, sphingomyelin; TAG, triacylglycerol.

Among the 36 known lipid species, 23 were inversely and 13 were positively associated with physical activity levels. The strongest positive associations were observed for long-chain and very-long-chain unsaturated PC plasmalogens, while the strongest inverse associations were observed for long-chain unsaturated triacylglycerols (TAGs) and ceramides (CERs) (Fig. 2; **Supplemental Table ****S1**). Notably, all the identified AC, CERs, FA, PEs, TAGs were inversely associated with habitual physical activity level, while the majority of the identified PCs or PC plasmalogens (12 out of 15) and 1 SM (SM (d39:1) B) were positively associated with physical activity levels.

### Physical activity-related lipids associated with incident diabetes/prediabetes

Among the 1,047 study participants who had normal fasting glucose at baseline and were followed through 2006–2009 (mean follow-up 5.5 years), 178 developed incident prediabetes and 68 developed incident diabetes. Overall, 18 out of the 36 physical activity-related lipids (q < 0.05, known) were associated with incident diabetes (Fig. 2; **Supplemental Table ****S1**) or combined incidence of diabetes/prediabetes (**Supplemental Figure ****S2****; Supplemental Table ****S1**).

Of the 23 lipids that showed an inverse association with physical activity levels, 16 were further linked to a higher risk of diabetes at *P* < .05. The strongest positive associations with diabetes risk were observed for PC(38:3) (OR: 1.71; 95% CI: 1.33, 2.21), followed by multiple long-chain unsaturated TAGs and PEs. In addition, one lipid [i.e., PE(36:1) A] was linked to a higher risk of prediabetes. On average, each standard deviation (SD) increase in the baseline level of these lipids was associated with 21%-71% increased risk of diabetes (from OR: 1.21; 95% CI: 1.01,1.44; to OR: 1.71; 95% CI: 1.33, 2.21).

Of the 13 lipids that showed a positive association with physical activity, no lipids reached statistical significance for the their associations with diabetes risk, while one PC plasmalogen [PC(p-18:1/20:4)/PC(o-18:2/20:4)] was linked to a lower risk of prediabetes, and two PC plasmalogens [PC(p-18:1/20:4)/PC(o-18:2/20:4) and PC(p-22:3/18:5)/PC(o-22:4/18:5)] were linked to a lower risk of combined incidence of diabetes/prediabetes. Each SD increase in the baseline level of the two PC plasmalogens was associated with 19%-22% lower risk of combined diabetes/prediabetes (OR: 0.81; 95% CI: 0.68, 0.96; and OR: 0.78; 95% CI: 0.66, 0.91). Overall, we observed similar associations when the outcome of interest was combined incident diabetes /prediabetes, or incident diabetes only.

### Physical activity-related lipids associated with glucose/insulin metrics

We included 1,277 participants who had complete data on glucose/insulin metrics at both baseline and follow-up examinations for this analysis. The majority (28 out of 36) of the q-value significant lipids were longitudinally associated with at least one of the glucose/insulin measures (Fig. 2; **Supplemental Table ****S2**).

Among the 23 lipids that were inversely associated with physical activity levels, 16 consistently showed positive associations with both fasting glucose and insulin resistance (HOMA-IR), as well as inverse associations with insulin sensitivity (QUICKI). This includes all but one CERs (4 lipids), all PEs (3 lipids), and all TAGs (9 lipids). Among the 13 lipids positively linked with physical activity levels, 6 showed positive associations with insulin sensitivity (QUICKI), while weak correlations with fasting glucose and insulin resistance were observed.

**Fig. 3:**
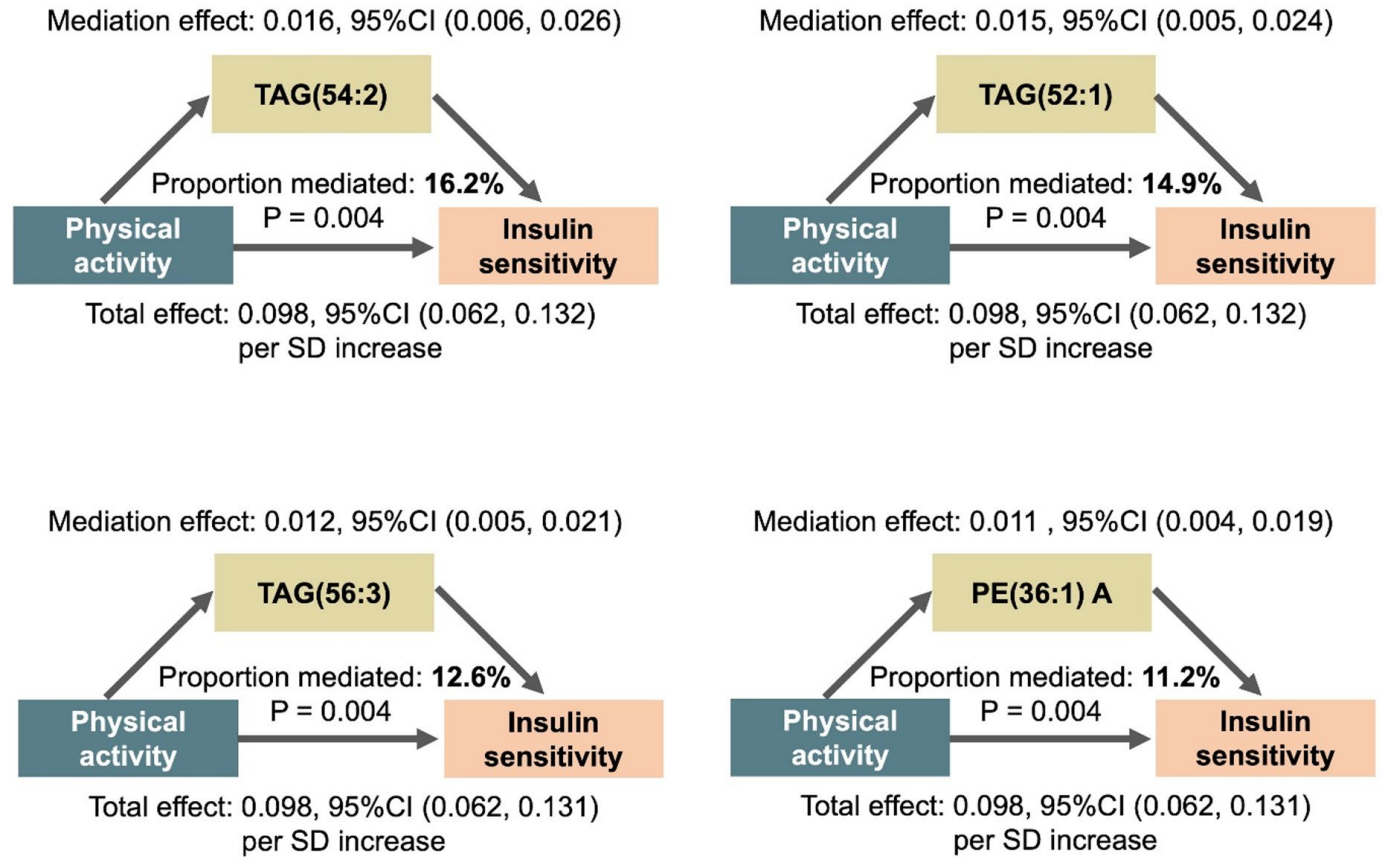
Lipids mediate the association between physical activity and insulin sensitivity. The top four lipids with the largest proportion of mediation were shown. The letter A at the end of lipid names indicates isomers. Covariates included age, sex, study center, education, BMI, smoking, alcohol use, lipid-lowering medication use and hypertension. Mediation percentage of lipids was calculated as the average causal mediation effects relative to the total effect. PE, phosphatidylethanolamine, SD, standard deviation; TAG, triacylglycerol.

Mediation analyses identified 28 lipids as significant mediators for the positive association of physical activity with insulin sensitivity (QUICKI), with proportion mediated range from 2.0% (95%CI: 0.04, 5.79) − 16.2% (95%CI: 6.88, 27.40). The top 4 lipids with the largest proportion mediated are shown in Fig. 3 (full results are shown in **Supplemental Table S3**). Notably, the above-mentioned 4 CERs, 3 PEs, and 9 TAGs that were consistently associated with glucose/insulin metrics all showed significant mediation effects on the physical activity-insulin sensitivity association. No mediation effects were found for physical activity-fasting glucose or -insulin resistance associations.

### Pathway enrichment analyses

Of the 128 lipids (known) associated with physical activity at *P* < .05, 94 were successfully matched to the Human Metabolome Database (HMDB) or the Kyoto Encyclopedia of Genes and Genomes (KEGG) database. The top significant pathways are glycerophospholipid metabolism and sphingolipid metabolism, with only glycerophospholipid metabolism remaining significant at FDR < 0.05 (**Supplemental Figure S3**).

### Lipidomic score of habitual physical activity

We constructed the lipidomic score based on the 36 known lipid species associated with physical activity at q < 0.05. The score significantly differed (ANOVA *P* < .05) across poor, intermediate and ideal physical activity status (defined as < 3,500, 3,500 − 10,000 and > 10,000 steps/day, respectively, based on previous SHFS reports^32^. The highest scores were observed in the ideal group, and the lowest in the poor group. The lipidomic score alone achieved an area under the ROC curve (AUC) of 0.802 (95%CI: 0.756–0.847) when discriminating poor vs. ideal status **(Supplemental Figure S4**).

After adjusting for BMI and other risk factors, the baseline lipidomic score for habitual physical activity was inversely associated with incident diabetes, and combined incidence of diabetes and prediabetes (**Supplemental Figure S5**). Each SD increase in the lipidomic score was associated with a 36% lower risk of diabetes (OR: 0.64; 95% CI: 0.498, 0.823). In addition, the score was associated with insulin resistance inversely, and insulin sensitivity positively (beta: -0.217; 95% CI: -0.273, -0.161 for HOMA-IR, and 0.258 (0.206, 0.310) for QUCKI).

### Results from sensitivity analyses

The sensitivity analyses included a subset of participants (*n* = 1,608 at baseline; *n* = 1,247 at follow-up) with available AHEI data. Among them, 949 participants had normal fasting glucose at baseline and were included in the analyses of associations with incident prediabetes and diabetes. After additionally adjusting for AHEI, the results remained largely unchanged. Most (> 70%) lipids remained significant in the sensitivity analyses, with associations with physical activity, prediabetes, and diabetes largely consistent with the main analyses (**Supplemental Table S4**).

## Discussion

Based on a longitudinal study design, we identified several key findings. First, we identified 36 lipidomic markers of habitual physical activity, primarily consisting of glycerophospholipids (e.g., phosphatidylcholines [PCs], PC plasmalogens, phosphatidylethanolamines [PEs]), triacylglycerols (TAGs), and ceramides (CERs), after adjusting for BMI and socio-demographic, lifestyle and clinical covariates. Second, half of these physical activity-related lipid species were significantly associated with the risk of developing diabetes or prediabetes over about a 5.5-year follow-up period. Third, these lipids were also associated with changes in glucose and insulin homeostasis over time, with several mediating the relationship between habitual physical activity and insulin sensitivity. Lastly, the derived lipidomic score reflected habitual physical activity levels and was associated with future diabetes risk. Collectively, these findings illuminate a mechanistic link between physical activity, lipid metabolism, and diabetes risk in American Indians – an understudied high-risk population. By identifying lipidomic signatures that are modifiable through lifestyle behaviors, this work highlights the potential of these biomarkers to serve as early indicators of metabolic vulnerability and actionable targets for precision prevention. Integrating lipidomic profiling into risk stratification frameworks could enable more timely, culturally tailored interventions that improve diabetes outcomes in this disproportionately affected community.

We found that long-chain and very-long-chain glycerophospholipids, particularly PCs and PC plasmalogens, are the predominant lipid classes associated with habitual physical activity, with PC plasmalogens exhibiting the strongest associations. Additionally, glycerophospholipid metabolism was one of the top over-represented pathways implicated in physical activity-related lipids. These findings align with previous studies. For example, in a study of 5,197 US participants^12^, 20 metabolites were found to be associated with self-reported physical activity, with PCs and lysophosphatidylcholine (LPCs) comprising the largest groups. Their analyses showed that physical activity was positively associated with the identified PC species, largely consistent with our findings. We also found that two PC plasmalogens [PC(p-18:1/20:4)/PC(o-18:2/20:4) and PC(p-22:3/18:5)/PC(o-22:4/18:5)] were linked to higher activity level and lower risk of combined diabetes and prediabetes. PCs and PC plasmalogens are key components of cell membranes. PCs are involved in maintaining membrane integrity, fluidity and energy metabolism^33^, and PC plasmalogens play key roles in cellular signaling, antioxidant defense^34^ and adipose tissue metabolism^35^, which may explain their prominence as markers of physical activity. Furthermore, we identified 3 PEs that were inversely associated with physical activity but positively with risk of diabetes or combined diabetes/prediabetes. Many of the identified PCs, PC plasmalogens and PEs partially mediated the association of physical activity with insulin sensitivity. These findings are in agreement with our previous lipidomic studies in the Strong Heart Study showing that disrupted glycerophospholipid metabolism was associated with risk of diabetes^18^ and other metabolic disorders such as hypertension^16^, coronary heart disease^15^ and chronic kidney disease^17^, and suggest that physical activity may affect diabetes risk through altering the metabolism of glycerophospholipids.

Triacylglycerols (TAGs) are the primary form of stored lipids in the body and play a central role in energy metabolism. During periods of inadequate physical activity or prolonged sedentary behavior, excess energy is stored as TAGs in adipose tissue. This may help explain why TAGs were the predominant lipid class inversely correlated with habitual physical activity in our study, exhibiting one of the strongest associations. Supporting our observations, prior metabolomics studies also reported inverse associations between TAGs and self-reported physical activity^11,12^. Similarly, physical activity improved insulin sensitivity and reduced circulating TAG levels^36,37^. Clinical trials have further demonstrated that physical activity interventions can effectively enhance insulin sensitivity in adults with overweight, obesity, or prediabetes^38,39^. We further demonstrated that these physical activity-related TAGs were strongly and consistently associated with an increased risk of diabetes. Additionally, these TAGs partially mediate the relationship between physical activity and insulin sensitivity, with predominant species being long-chain TAGs of low to moderate unsaturation. A previous study^40^ identified multiple long-chain unsaturated TAG species in adipose tissue that differed significantly among insulin-sensitive, insulin-resistant, and diabetic individuals, including TAG(56:3), which was a significant mediator of insulin sensitivity in our study. Importantly, consistent with our findings, their observations were also independent of BMI. This concordance suggests a potential link between TAG metabolism at both the blood and tissue levels and insulin sensitivity, which could to be influenced by physical activity independently of BMI.

We observed that several long-chain and very-long-chain ceramides (CERs) were inversely associated with physical activity levels while being positively correlated with incident diabetes and deterioration of glucose metabolism over time. CERs are essential components of cell membranes and play crucial roles in cell signaling and inflammation regulation. Elevated CER levels have been linked to impaired insulin sensitivity, inflammation, and oxidative stress^41,42^, all of which are known to be involved in the development of diabetes. A previous study conducted in the Strong Heart Study found that higher circulating levels of certain CERs were associated with an increased risk of diabetes in American Indian adults^43^. Another study reported elevated CER concentrations in diabetes patients compared to healthy controls, which correlated with greater insulin resistance and higher levels of inflammatory marker TNF-α levels^44^. Our finding that long-chain and very-long-chain CERs are major lipid mediators of insulin sensitivity also aligns with evidence from animal studies. In one study of mouse muscle tissue, multiple very-long-chain CERs were highly associated with insulin resistance. Importantly, this association was independent of fat mass, similar to our BMI-independent associations between CERs and insulin sensitivity and resistance. Together, these results suggest that lipid profiles could provide a link between physical activity and systemic insulin action, independent of BMI or obesity^45^.

Evidence from a large body of clinical trials has shown that physical activity improves insulin sensitivity^46^. We extend these findings by demonstrating that the majority of physical activity-related lipids (e.g., specific PCs, PEs, plasmalogens, TAGs and CERs) may lie on the mediating pathway between habitual physical activity and insulin sensitivity. Furthermore, a lipidomic score derived from the identified lipids reflects habitual physical activity levels and is inversely associated with future diabetes risk. If validated, our findings could offer new insights into the biological mechanisms underlying the effects of physical activity and support early intervention and personalized strategies for diabetes prevention.

One of the key strengths of this study is its longitudinal study design in a well-characterized cohort. The longitudinal nature of the cohort and temporal sequencing of exposure, lipidomic profiling, and T2D onset strengthen our ability to detect predictive relationships within a biomarker discovery framework. Moreover, the extensive lipidomic coverage allows for a comprehensive evaluation of lipid markers associated with physical activity. The use of pedometers to objectively quantify physical activity levels reduces recall bias commonly associated with self-reported data. Furthermore, the prospective cohort design allows us to evaluate the relevance of these lipidomic markers in relation to the risk of diabetes and prediabetes, as well as changes in diabetes risk over time.

Several limitations should also be acknowledged. First, among the over 1,500 detected lipids, many are unknown compounds and will require further characterization in future studies. Second, the pedometer-based measures capture only ambulatory activity and do not account for non-ambulatory physical activities, such as swimming or upper body exercises. Third, lipid levels were measured using single time-point samples at both baseline and follow-up, which may not fully reflect biological variation in lipid levels. Fourth, as our study focuses on American Indians, future research in diverse cohorts is warranted to assess the generalizability of these findings. Lastly, as this study is observational, it is primarily intended for biomarker discovery, and causal inferences cannot be drawn.

In summary, habitual physical activity was associated with significant alterations in lipidomic profile in American Indian adults. These lipidomic markers, individually or in combination, were linked to diabetes and measures of glucose and insulin homeostasis, above and over clinical covariates. These associations may be partly explained by the mediating role of some specific lipids in the relationship between physical activity and insulin sensitivity. Future studies are warranted to validate these findings in diverse populations and to further elucidate the underlying mechanisms.

## Supplementary Information

Below is the link to the electronic supplementary material.

Supplementary Material 1

Supplementary Material 2

## Data Availability

The phenotype data used in this study can be requested through the Strong Heart Study (https://strongheart-study.org/). The lipidomic data can be obtained from the corresponding author upon a reasonable request.

## Abbreviations

AC: Acylcarnitine
BCAA: Branched-chain amino acids
CE: Cholesterol ester
CER: Ceramides
FDR: False discovery rate
HMDB: Human Metabolome Database
KEGG: Kyoto Encyclopedia of Genes and Genomes
LPC: Lysophosphatidylcholine
PE: Phosphatidylethanolamine
PC: Phosphatidylcholine
SD: Standard deviation
SHFS: Strong Heart Family Study
SM: Sphingomyelin
TAG: Triacylglycerol
