# Supplementary material for: Lipidomic markers of habitual physical activity and risk of type 2 diabetes in American Indians
Source: Sci Rep. 2025 Nov 24;15:45228. doi: 10.1038/s41598-025-29211-y (PMC12749243; doi:10.1038/s41598-025-29211-y)
Supplement: Supplementary file 1 — Supplementary Material 1 [file 41598_2025_29211_MOESM1_ESM.docx]

**Lipidomic Markers of Habitual Physical Activity and Risk of Type 2 Diabetes in American Indians. Wen X, et al.**

**Supplemental Methods**

**Blood sample collection**

Participants were instructed to fast overnight before their visit, and fasting blood sample was collected into 10ml EDTA tubes at the Strong Heart Study field centers. The tubes were then gently inverted and placed on ice or refrigerated (- 4 °C) immediately. Plasma sample was obtained by centrifuging the tubes for 10 minutes at 3,000 rpm at - 4 °C and aliquots (0.5 ml) were immediately stored at -80 °C until further analysis. For the current study, 0.5 ml fasting plasma sample, which were never thawed before, was shipped to Dr. Fiehn’s lab at the West Coast Metabolomics Center (UC-Davis) on dry ice via FedEx overnight, and stored at -80 °C immediately on arrival until further analyses. Samples were randomized before shipping to the Fiehn’s laboratory, where randomization was performed again before the lipidomics analysis as described below.

**Lipidomic data acquisition**

Per our previously published protocols (1), we used the following detailed methods for lipid quantification and lipid annotations.

The LC–MS systems used and injection volumes are given here:

| LC system | MS system | R (fwhm) | resuspension volume (μL) | injection volume (μL) |
| --- | --- | --- | --- | --- |
| Agilent 1290 Infinity | Agilent 6530 | 10,000 | 200 | 1.5 |
| Agilent 1290 Infinity | Agilent 6550 iFunnel | 20,000 | 500 | 1.5 |

Each LC system consisted of a pump, a column oven, and an autosampler. Lipids were separated on an Acquity UPLC CSH C18 column (100 × 2.1 mm; 1.7 μm) coupled to an Acquity UPLC CSH C18 VanGuard precolumn (5 × 2.1 mm; 1.7 μm) (Waters, Milford, MA). The column was maintained at 65 °C at a flow-rate of 0.6 mL/min. The mobile phases consisted of (A) 60:40 (v/v) acetonitrile:water with ammonium formate (10 mM) and formic acid (0.1%) and (B) 90:10 (v/v) isopropanol:acetonitrile with ammonium formate (10 mM) and formic acid (0.1%). The separation was conducted under the following gradient: 0 min 15% (B); 0–2 min 30% (B); 2–2.5 min 48% (B); 2.5–11 min 82% (B); 11–11.5 min 99% (B); 11.5–12 min 99% (B); 12–12.1 min 15% (B); and 12.1–15 min 15% (B). Sample temperature was maintained at 4 °C. Detailed instrumental parameters are described here:

*Agilent 6530 QTOF MS*

Analyses on both quadrupole/time-of-flight mass spectrometers Agilent 6530 with a Dual Spray ESI ion source (Agilent Technologies, Santa Clara, CA) were performed at the high sensitivity mode. Simultaneous MS1 and MS/MS (All Ion MS/MS) acquisition was used. The parameters were ESI polarity, positive; capillary voltage, 3.5 kV; nozzle voltage, 1 kV; gas temperature, 325 °C; drying gas (nitrogen), 8 L/min; nebulizer gas (nitrogen), 35 psi; sheath gas temperature, 350 °C; sheath gas flow (nitrogen), 11 L/min; MS1 acquisition speed, 2 spectra/s; MS1 mass range, *m*/*z* 60–1700; MS/MS acquisition speed, 2 spectra/s; MS/MS mass range, *m*/*z* 60–1700; collision energy, 25 eV. The instrument was tuned using an Agilent tune mix. A reference solution (*m*/*z* 121.0509, *m*/*z* 922.0098) was used to correct small mass drifts during the acquisition.

*Agilent 6550 iFunnel QTOF MS*

Analyses on an Agilent 6550 iFunnel QTOF with a Dual Spray ESI ion source (Agilent Technologies) were performed at the high sensitivity mode. Simultaneous MS1 and MS/MS (All Ion MS/MS) acquisition was used. The parameters were ESI polarity, positive; capillary voltage, 3.5 kV; nozzle voltage, 1 kV; gas temperature, 200 °C; drying gas (nitrogen), 14 L/min; nebulizer gas (nitrogen), 35 psi; sheath gas temperature, 350 °C; sheath gas flow (nitrogen), 11 L/min; MS1 acquisition speed, 2 spectra/s; MS1 mass range, *m*/*z* 60–1700; MS/MS acquisition speed, 2 spectra/s; MS/MS mass range, *m*/*z* 60–1700; collision energy, 25 eV. The instrument was tuned using an Agilent tune mix. A reference solution (*m*/*z* 121.0509, *m*/*z* 922.0098) was used to correct small mass drifts during the acquisition.

The following internal standards were used for both correction of small metabolite drifts and as surrogates for quantifications:

| **Concentrations (nmol/mL) of the Internal Standards Spiked in Blood Plasma**  internal standard *a* | concentration  (nmol/mL plasma) | used for retention time correction | used for quantification |
| --- | --- | --- | --- |
| CUDA | 150b | ✓ | 🗶 |
| Sphingosine (d17:1) | 17.9 | ✓ | 🗶 |
| LPE(17:1) | 49.3 | ✓ | ✓ |
| LPC(17:0) | 30 | ✓ | ✓ |
| MG(17:0/0:0/0:0) | 178 | ✓ | 🗶 |
| DG(18:1/2:0/0:0) | 230 | ✓ | 🗶 |
| PC(12:0/13:0) | 1.44 | ✓ | ✓ |
| DG(12:0/12:0/0:0) | 101 | ✓ | ✓ |
| *d7-Cholesterol* | 155 | ✓ | ✓ |
| SM(d18:1/17:0) | 8.54 | ✓ | ✓ |
| PG(17:0/17:0) | 57.7 | ✓ | 🗶 |
| Cer(d18:1/17:0) | 13.9 | ✓ | ✓ |
| PE(17:0/17:0) | 16 | ✓ | ✓ |
| *d*5-TG(17:0/17:1/17:0) | 6.74 | ✓ | ✓ |
| CE(22:1) | 771 | ✓ | ✓ |

Overall precision achieved was analyzed by plasma pool quality controls, obtained from Bioreclamation (now BioIVT) company, with the following coefficient of variance:

|  | biorec %RSD | NIST %RSD |
| --- | --- | --- |
| internal standards | 3.3% | 10.1% |
| known compounds | 4.3% | 12.6% |
| unknown compounds | 12.8% | 19.6% |

No drift was observed. A total of 207 Bioreclamation pool plasma quality controls were analyzed, along with a total of 54 NIST SRM1950 pool plasma quality controls.

**Quality Control**

Quality control was assured by (i) randomization of the sequence, (ii) injection of 10 pool samples to equilibrate the LC–MS system before actual sequence of samples; (iii) injection of pool samples at the beginning and the end of the sequence and between each 10 actual samples, (iv) injection of NIST SRM 1950 at the beginning of the sequence and after injection of 100 actual samples; (v) procedure blank analysis, (vi) checking the peak shape and the intensity of spiked internal standards and the internal standard added prior to injection, and (vii) monitoring mass accuracy of internal standards during the run. Laboratory technicians were blinded to all clinical data throughout the assays.

**Lipidomics data processing and normalization**

The lipidomics data were pre-processed using a new in-house cloud-based software (LC-BinBase) with peak detection and deconvolution algorithms adapted from MS-DIAL (2). Raw files were automatically converted into correct formats and the LC-BinBase algorithms performed peak picking, retention time alignment using internal standards, and gap filling from raw data for missed peaks. Lipid peak intensity results were manually checked against raw data files. Adducts were combined into single features for statistical assessments. False negative features and peaks with 50% missing values across all samples were removed. The batch effect of reformatted dataset was normalized by SERRF software (Systematic Error Removal using Random Forest) (3), which dramatically reduced the raw data variance coefficient by 23% in positive mode data and 25% in negative mode to less than 10% in result files. The lipid levels of the duplicated samples were highly correlated (Spearman’s correlation coefficient 𝜌 =0.95, p<2.2× 10^−16^ ). Outlier samples were detected by principal component analysis, and those beyond mean ± 5 SD for any of the first three PCs were further removed. The resulting lipidomics data were standardized to zero mean and unit variance prior to statistical analyses.

**Interpolating missing values**

For alignment IDs that passed the filtering process but still contained missing values, MS-DIAL applied an interpolation method. First, the software calculated the average retention time and average m/z (mass-to-charge ratio) from the “filled” peaks, providing a reference point based on detected signals. Next, the software scanned within a defined range around the average retention time and m/z to identify the local maximum intensity value, which was then used to fill in the missing values. This process ensured that missing values were estimated based on the nearest available data points. After preprocessing and quality control, we obtained 1,542 lipids (518 known, 1,024 unknown) in 1,957 participants at baseline, and 1,948 participants at 5-year follow-up. The lipid classes include: Acylcarnitine (AC), Cholesterol ester (CE), Ceramide (CER), Cholesterol, Diacylglycerol (DAG), Fatty acid (FA), Glycosylceramide (GlcCer), Phosphatidylcholine (PC), Phosphatidylethanolamine (PE), Phosphatidylglycerol (PG), Phosphatidylinositol (PI), Phosphatidylserine (PS), Sphingomyelin (SM), Triacylglycerol (TAG).

**Reference:**

1. Cajka T, Smilowitz JT, Fiehn O. Validating Quantitative Untargeted Lipidomics Across Nine Liquid Chromatography-High-Resolution Mass Spectrometry Platforms. Anal Chem. 2017;89(22):12360-8.
