# Supplementary material for: Lipidomic markers of habitual physical activity and risk of type 2 diabetes in American Indians
Source: Sci Rep. 2025 Nov 24;15:45228. doi: 10.1038/s41598-025-29211-y (PMC12749243; doi:10.1038/s41598-025-29211-y)
Supplement: Supplementary file 2 — Supplementary Material 2 [file 41598_2025_29211_MOESM2_ESM.docx]

**Lipidomic Markers of Habitual Physical Activity and Risk of Type 2 Diabetes in American Indians. Wen X, et al.**

**Supplemental Tables and Figures**

**Table of Contents**

**Supplemental Table S1.** Plasma lipid species associated with physical activity levels (q<0.05) and diabetes risk

**Supplemental Table S2.** Physical activity-related lipids (q<0.05) associated with glucose/insulin homeostasis metrics

**Supplemental Table S3.** Mediation effects of lipids on the physical activity-Insulin sensitivity association (N = 1,277)

**Supplemental Table S4.** Plasma lipid species associated with physical activity levels (q<0.05) and diabetes risk in sensitivity analyses

**Supplemental Figure S1.** Participant selection and analyses flowchart

**Supplemental Figure S2.** Plasma lipid species associated with physical activity levels (q<0.05), incident prediabetes and combined incidence of T2D/prediabetes

**Supplemental Figure S3.** Pathway enrichment results for physical activity-related lipids (P<0.05)

**Supplemental Figure S4.** Discriminative ability of the lipidomic score

**Supplemental Figure S5.** Baseline lipidomic score is associated with diabetes risk

**Supplemental Table S1.** Plasma lipid species associated with physical activity levels (q<0.05) and diabetes risk

| **Lipids** | **Class** | **Association with physical activity** | |  | **Associations with prediabetes or T2D, OR(95%)^3^** | | |
| --- | --- | --- | --- | --- | --- | --- | --- |
|  |  | **Beta (95%)^1^** | **q value²** |  | **T2D** | **Prediabetes** | **Prediabetes/T2D** |
| AC(18:1) B | Acylcarnitine | **-0.04 (-0.08,-0.01)** | 0.05 |  | 0.87 (0.67,1.13) | 1.10 (0.94,1.29) | 1.03 (0.89,1.20) |
| CER(d34:0) | Ceramide | **-0.05 (-0.08,-0.02)** | 0.037 |  | **1.28 (1.04,1.58)** | 1.07 (0.91,1.26) | **1.19 (1.02,1.38)** |
| CER(d34:1) | Ceramide | **-0.08 (-0.11,-0.04)** | 0.001 |  | **1.40 (1.10,1.79)** | 1.01 (0.85,1.20) | 1.15 (0.98,1.34) |
| CER(d34:2) | Ceramide | **-0.06 (-0.10,-0.03)** | 0.008 |  | 1.20 (0.93,1.56) | 0.85 (0.71,1.02) | 0.94 (0.80,1.11) |
| CER(d36:1) | Ceramide | **-0.08 (-0.11,-0.05)** | <0.001 |  | **1.37 (1.08,1.75)** | 1.05 (0.89,1.25) | **1.19 (1.01,1.39)** |
| CER(d42:2) A | Ceramide | **-0.06 (-0.09,-0.02)** | 0.022 |  | **1.34 (1.05,1.71)** | 1.03 (0.87,1.22) | 1.15 (0.98,1.34) |
| FA(16:0) | Fatty acid | **-0.04 (-0.08,-0.01)** | 0.046 |  | 1.05 (0.79,1.40) | 0.97 (0.78,1.21) | 0.99 (0.82,1.19) |
| LPC(20:1) | Lysophosphatidylcholine | **-0.05 (-0.08,-0.02)** | 0.037 |  | 1.10 (0.84,1.44) | 0.94 (0.78,1.14) | 0.99 (0.84,1.17) |
| LPC(22:6) | Lysophosphatidylcholine | **0.06 ( 0.03, 0.10)** | 0.003 |  | 0.90 (0.64,1.25) | 0.92 (0.76,1.12) | 0.90 (0.76,1.08) |
| PC(20:2/20:2) | Phosphatidylcholine | **0.05 ( 0.02, 0.08)** | 0.035 |  | 1.04 (0.82,1.32) | 1.07 (0.92,1.25) | 1.07 (0.93,1.24) |
| PC(37:4) | Phosphatidylcholine | **0.05 ( 0.02, 0.09)** | 0.037 |  | 1.07 (0.83,1.37) | 0.84 (0.68,1.03) | 0.90 (0.76,1.07) |
| PC(38:3) | Phosphatidylcholine | **-0.05 (-0.08,-0.02)** | 0.037 |  | **1.71 (1.33,2.21)** | 1.06 (0.89,1.27) | **1.28 (1.10,1.50)** |
| PC(38:6) A | Phosphatidylcholine | **0.06 ( 0.02, 0.09)** | 0.026 |  | 1.03 (0.77,1.38) | 0.94 (0.78,1.14) | 0.96 (0.81,1.14) |
| PC(40:5) B | Phosphatidylcholine | **-0.05 (-0.08,-0.01)** | 0.05 |  | **1.32 (1.04,1.69)** | 0.98 (0.83,1.17) | 1.11 (0.95,1.30) |
| PC(p-16:0/22:6)/PC(o-16:1/22:6) | Ether-phosphatidylcholine | **0.08 ( 0.04, 0.11)** | <0.001 |  | 1.07 (0.82,1.38) | 0.91 (0.76,1.09) | 0.94 (0.80,1.11) |
| PC(p-18:1/20:4)/PC(o-18:2/20:4) | Ether-phosphatidylcholine | **0.08 ( 0.05, 0.12)** | <0.001 |  | 0.89 (0.68,1.17) | **0.77 (0.65,0.93)** | **0.78 (0.66,0.91)** |
| PC(p-18:2/17:0)/PC(o-18:3/17:0) | Ether-phosphatidylcholine | **0.05 ( 0.02, 0.09)** | 0.027 |  | 0.95 (0.70,1.30) | 1.00 (0.83,1.21) | 0.99 (0.84,1.18) |
| PC(p-22:3/18:5)/PC(o-22:4/18:5) | Ether-phosphatidylcholine | **0.05 ( 0.02, 0.09)** | 0.029 |  | 0.81 (0.60,1.09) | 0.84 (0.70,1.02) | **0.81 (0.68,0.96)** |
| PC(p-34:2)/PC(o-34:3) | Ether-phosphatidylcholine | **0.06 ( 0.02, 0.09)** | 0.019 |  | 1.15 (0.87,1.52) | 0.97 (0.80,1.17) | 1.04 (0.88,1.22) |
| PC(p-36:4)/PC(o-36:5) | Ether-phosphatidylcholine | **0.05 ( 0.02, 0.09)** | 0.036 |  | 1.14 (0.86,1.52) | 0.96 (0.79,1.16) | 1.01 (0.85,1.19) |
| PC(p-36:5)/PC(o-36:6) | Ether-phosphatidylcholine | **0.06 ( 0.02, 0.09)** | 0.026 |  | 1.13 (0.86,1.48) | 1.00 (0.84,1.20) | 1.03 (0.88,1.22) |
| PC(p-38:5)/PC(o-38:6) B | Ether-phosphatidylcholine | **0.05 ( 0.02, 0.09)** | 0.027 |  | 1.06 (0.80,1.39) | 0.90 (0.74,1.08) | 0.93 (0.79,1.10) |
| PE(18:0/22:5) A | Phosphatidylethanolamine | **-0.05 (-0.08,-0.01)** | 0.045 |  | **1.44 (1.15,1.80)** | 1.04 (0.88,1.23) | **1.20 (1.03,1.40)** |
| PE(34:1) | Phosphatidylethanolamine | **-0.05 (-0.09,-0.02)** | 0.027 |  | **1.49 (1.20,1.86)** | 1.12 (0.95,1.32) | **1.30 (1.12,1.51)** |
| PE(36:1) A | Phosphatidylethanolamine | **-0.05 (-0.08,-0.01)** | 0.047 |  | **1.49 (1.22,1.82)** | **1.18 (1.01,1.38)** | **1.39 (1.20,1.62)** |
| SM(d39:1) B | Sphingomyelin | **0.07 ( 0.04, 0.11)** | 0.003 |  | 1.04 (0.77,1.39) | 0.94 (0.78,1.14) | 0.96 (0.81,1.14) |
| SM(d44:2) A | Sphingomyelin | **-0.05 (-0.08,-0.01)** | 0.045 |  | **1.42 (1.10,1.83)** | 1.03 (0.86,1.23) | 1.16 (0.99,1.36) |
| TAG(50:2) | Triacylglycerol | **-0.05 (-0.09,-0.02)** | 0.037 |  | **1.59 (1.22,2.08)** | 1.18 (0.99,1.41) | **1.35 (1.15,1.58)** |
| TAG(50:3) A | Triacylglycerol | **-0.05 (-0.08,-0.01)** | 0.045 |  | **1.41 (1.11,1.79)** | 1.08 (0.91,1.28) | **1.21 (1.04,1.40)** |
| TAG(52:1) | Triacylglycerol | **-0.05 (-0.08,-0.02)** | 0.042 |  | **1.58 (1.28,1.94)** | 1.16 (0.99,1.36) | **1.38 (1.19,1.60)** |
| TAG(53:3) | Triacylglycerol | **-0.05 (-0.08,-0.01)** | 0.048 |  | **1.27 (1.02,1.57)** | 1.07 (0.90,1.27) | **1.16 (1.00,1.35)** |
| TAG(54:2) | Triacylglycerol | **-0.06 (-0.10,-0.03)** | 0.009 |  | **1.36 (1.11,1.67)** | 1.15 (0.98,1.35) | **1.28 (1.11,1.48)** |
| TAG(54:3) | Triacylglycerol | **-0.07 (-0.10,-0.03)** | 0.005 |  | 1.16 (0.90,1.49) | 1.09 (0.92,1.30) | 1.13 (0.97,1.32) |
| TAG(54:4) | Triacylglycerol | **-0.05 (-0.09,-0.02)** | 0.028 |  | 1.04 (0.79,1.36) | 0.99 (0.83,1.19) | 1.00 (0.85,1.17) |
| TAG(55:4)/TAG(18:1/18:2/19:1) | Triacylglycerol | **-0.05 (-0.08,-0.01)** | 0.05 |  | **1.21 (1.01,1.44)** | 1.02 (0.86,1.21) | 1.11 (0.96,1.27) |
| TAG(56:3) | Triacylglycerol | **-0.06 (-0.09,-0.02)** | 0.022 |  | 1.18 (0.99,1.41) | 1.06 (0.90,1.24) | 1.13 (0.98,1.29) |

The letter A, B or C at the end of lipid names indicates isomers. Boldface indicates statistical significance.

¹ Beta coefficient of lipids associated with habitual physical activity. Obtained by mixed-effects linear regression model, adjusting for age, sex, study center, education, BMI, fasting glucose, smoking, alcohol use, lipid-lowering medication use, and hypertension status at the study visit. Random effects were used to account for family relatedness and repeated measurements at baseline and follow-up visits.

² Adjusted for multiple testing using the Storey’s q-value method.

^3^ Odds ratio of baseline lipids associated with incident outcomes. Obtained by mixed-effects logistic models, adjusting for all covariates above (baseline level).

**Supplemental Table S2.** Physical activity-related lipids (q<0.05) associated with glucose/insulin homeostasis metrics

| **Lipids** | **Class** | **Association with physical activity** | |  | **Associations with glucose/insulin homeostasis metrics, beta(95%CI)^3^** | | |
| --- | --- | --- | --- | --- | --- | --- | --- |
|  |  | **Beta (95%CI)¹** | **q value²** |  | **Fasting glucose** | **HOMA-IR** | **QUCKI** |
| AC(18:1) B | Acylcarnitine | **-0.04 (-0.08,-0.01)** | 0.05 |  | 0.00 (-0.03, 0.04) | **-0.07 (-0.11,-0.03)** | **0.09 ( 0.06, 0.12)** |
| CER(d34:0) | Ceramide | **-0.05 (-0.08,-0.02)** | 0.037 |  | **0.20 ( 0.17, 0.23)** | **0.08 ( 0.05, 0.12)** | **-0.13 (-0.16,-0.10)** |
| CER(d34:1) | Ceramide | **-0.08 (-0.11,-0.04)** | 0.001 |  | **0.16 ( 0.12, 0.19)** | **0.07 ( 0.04, 0.11)** | **-0.11 (-0.14,-0.07)** |
| CER(d34:2) | Ceramide | **-0.06 (-0.10,-0.03)** | 0.008 |  | 0.02 (-0.02, 0.06) | -0.02 (-0.06, 0.02) | 0.02 (-0.02, 0.05) |
| CER(d36:1) | Ceramide | **-0.08 (-0.11,-0.05)** | <0.001 |  | **0.11 ( 0.07, 0.14)** | **0.05 ( 0.01, 0.09)** | **-0.10 (-0.14,-0.07)** |
| CER(d42:2) A | Ceramide | **-0.06 (-0.09,-0.02)** | 0.022 |  | **0.07 ( 0.04, 0.11)** | **0.05 ( 0.01, 0.09)** | **-0.04 (-0.08,-0.01)** |
| FA(16:0) | Fatty acid | **-0.04 (-0.08,-0.01)** | 0.046 |  | -0.01 (-0.04, 0.03) | -0.01 (-0.05, 0.02) | -0.01 (-0.04, 0.02) |
| LPC(20:1) | Lysophosphatidylcholine | **-0.05 (-0.08,-0.02)** | 0.037 |  | **-0.05 (-0.08,-0.01)** | **-0.04 (-0.08, 0.00)** | **0.09 ( 0.05, 0.12)** |
| LPC(22:6) | Lysophosphatidylcholine | **0.06 ( 0.03, 0.10)** | 0.003 |  | 0.01 (-0.02, 0.04) | -0.01 (-0.05, 0.02) | **0.04 ( 0.01, 0.07)** |
| PC(20:2/20:2) | Phosphatidylcholine | **0.05 ( 0.02, 0.08)** | 0.035 |  | 0.03 ( 0.00, 0.07) | **0.04 ( 0.01, 0.08)** | **-0.04 (-0.07,-0.01)** |
| PC(37:4) | Phosphatidylcholine | **0.05 ( 0.02, 0.09)** | 0.037 |  | 0.03 (-0.01, 0.07) | **-0.04 (-0.09, 0.00)** | **0.10 ( 0.07, 0.14)** |
| PC(38:3) | Phosphatidylcholine | **-0.05 (-0.08,-0.02)** | 0.037 |  | **-0.05 (-0.08,-0.01)** | **0.06 ( 0.02, 0.10)** | **-0.14 (-0.18,-0.11)** |
| PC(38:6) A | Phosphatidylcholine | **0.06 ( 0.02, 0.09)** | 0.026 |  | 0.03 ( 0.00, 0.07) | -0.01 (-0.04, 0.03) | 0.03 ( 0.00, 0.07) |
| PC(40:5) B | Phosphatidylcholine | **-0.05 (-0.08,-0.01)** | 0.05 |  | 0.02 (-0.02, 0.06) | **0.04 ( 0.01, 0.08)** | **-0.09 (-0.13,-0.06)** |
| PC(p-16:0/22:6)/PC(o-16:1/22:6) | Ether-phosphatidylcholine | **0.08 ( 0.04, 0.11)** | <0.001 |  | 0.04 ( 0.00, 0.07) | **0.05 ( 0.02, 0.09)** | -0.03 (-0.06, 0.00) |
| PC(p-18:1/20:4)/PC(o-18:2/20:4) | Ether-phosphatidylcholine | **0.08 ( 0.05, 0.12)** | <0.001 |  | **-0.04 (-0.08,-0.01)** | -0.02 (-0.06, 0.02) | **0.08 ( 0.04, 0.11)** |
| PC(p-18:2/17:0)/PC(o-18:3/17:0) | Ether-phosphatidylcholine | **0.05 ( 0.02, 0.09)** | 0.027 |  | -0.02 (-0.06, 0.01) | 0.04 ( 0.00, 0.07) | 0.00 (-0.03, 0.03) |
| PC(p-22:3/18:5)/PC(o-22:4/18:5) | Ether-phosphatidylcholine | **0.05 ( 0.02, 0.09)** | 0.029 |  | -0.02 (-0.06, 0.01) | -0.02 (-0.06, 0.02) | **0.08 ( 0.04, 0.11)** |
| PC(p-34:2)/PC(o-34:3) | Ether-phosphatidylcholine | **0.06 ( 0.02, 0.09)** | 0.019 |  | **-0.05 (-0.09,-0.01)** | 0.01 (-0.03, 0.05) | **0.06 ( 0.03, 0.09)** |
| PC(p-36:4)/PC(o-36:5) | Ether-phosphatidylcholine | **0.05 ( 0.02, 0.09)** | 0.036 |  | 0.02 (-0.02, 0.06) | 0.03 (-0.01, 0.07) | 0.03 ( 0.00, 0.07) |
| PC(p-36:5)/PC(o-36:6) | Ether-phosphatidylcholine | **0.06 ( 0.02, 0.09)** | 0.026 |  | 0.00 (-0.03, 0.04) | 0.04 ( 0.00, 0.08) | -0.03 (-0.06, 0.01) |
| PC(p-38:5)/PC(o-38:6) B | Ether-phosphatidylcholine | **0.05 ( 0.02, 0.09)** | 0.027 |  | -0.01 (-0.04, 0.03) | -0.02 (-0.05, 0.02) | **0.06 ( 0.03, 0.10)** |
| PE(18:0/22:5) A | Phosphatidylethanolamine | **-0.05 (-0.08,-0.01)** | 0.045 |  | **0.11 ( 0.07, 0.14)** | **0.08 ( 0.04, 0.12)** | **-0.13 (-0.16,-0.10)** |
| PE(34:1) | Phosphatidylethanolamine | **-0.05 (-0.09,-0.02)** | 0.027 |  | **0.16 ( 0.12, 0.19)** | **0.10 ( 0.06, 0.14)** | **-0.14 (-0.17,-0.10)** |
| PE(36:1) A | Phosphatidylethanolamine | **-0.05 (-0.08,-0.01)** | 0.047 |  | **0.19 ( 0.16, 0.23)** | **0.13 ( 0.10, 0.17)** | **-0.17 (-0.20,-0.14)** |
| SM(d39:1) B | Sphingomyelin | **0.07 ( 0.04, 0.11)** | 0.003 |  | -0.03 (-0.08, 0.01) | -0.02 (-0.06, 0.02) | -0.01 (-0.04, 0.03) |
| SM(d44:2) A | Sphingomyelin | **-0.05 (-0.08,-0.01)** | 0.045 |  | 0.00 (-0.04, 0.04) | -0.02 (-0.06, 0.02) | 0.01 (-0.02, 0.05) |
| TAG(50:2) | Triacylglycerol | **-0.05 (-0.09,-0.02)** | 0.037 |  | **0.15 ( 0.11, 0.19)** | **0.12 ( 0.08, 0.16)** | **-0.20 (-0.24,-0.17)** |
| TAG(50:3) A | Triacylglycerol | **-0.05 (-0.08,-0.01)** | 0.045 |  | **0.16 ( 0.12, 0.19)** | **0.11 ( 0.07, 0.15)** | **-0.19 (-0.22,-0.16)** |
| TAG(52:1) | Triacylglycerol | **-0.05 (-0.08,-0.02)** | 0.042 |  | **0.21 ( 0.17, 0.24)** | **0.18 ( 0.14, 0.21)** | **-0.23 (-0.27,-0.20)** |
| TAG(53:3) | Triacylglycerol | **-0.05 (-0.08,-0.01)** | 0.048 |  | **0.23 ( 0.20, 0.27)** | **0.11 ( 0.07, 0.15)** | **-0.16 (-0.19,-0.13)** |
| TAG(54:2) | Triacylglycerol | **-0.06 (-0.10,-0.03)** | 0.009 |  | **0.23 ( 0.19, 0.27)** | **0.17 ( 0.13, 0.21)** | **-0.23 (-0.27,-0.20)** |
| TAG(54:3) | Triacylglycerol | **-0.07 (-0.10,-0.03)** | 0.005 |  | **0.17 ( 0.14, 0.21)** | **0.12 ( 0.08, 0.16)** | **-0.15 (-0.18,-0.12)** |
| TAG(54:4) | Triacylglycerol | **-0.05 (-0.09,-0.02)** | 0.028 |  | **0.17 ( 0.13, 0.20)** | **0.10 ( 0.06, 0.14)** | **-0.13 (-0.16,-0.10)** |
| TAG(55:4)/TAG(18:1/18:2/19:1) | Triacylglycerol | **-0.05 (-0.08,-0.01)** | 0.05 |  | **0.20 ( 0.16, 0.23)** | **0.08 ( 0.04, 0.11)** | **-0.12 (-0.16,-0.09)** |
| TAG(56:3) | Triacylglycerol | **-0.06 (-0.09,-0.02)** | 0.022 |  | **0.23 ( 0.20, 0.27)** | **0.13 ( 0.09, 0.17)** | **-0.19 (-0.22,-0.16)** |

The letter A, B or C at the end of lipid names indicates isomers. Boldface indicates statistical significance.

¹ Beta coefficient of lipids associated with habitual physical activity. Obtained by mixed-effects linear regression model, adjusting for age, sex, study center, education, BMI, fasting glucose, smoking, alcohol use, lipid-lowering medication use, and hypertension status at the study visit. Random effects were used to account for family relatedness and repeated measurements at baseline and follow-up visits.

² Adjusted for multiple testing using the Storey’s q-value method.

^3^ Beta coefficient of lipids associated with glucose/insulin metrics. Obtained by mixed-effects linear regression model, adjusting for the same covariates above, except fasting glucose. Random effects were used to account for family relatedness and repeated measurements at baseline and follow-up visits.

**Supplemental Table S3.** Mediation effects of lipids on the physical activity-Insulin sensitivity association (N = 1,277)

| **Lipids** | **Class** | **Association with physical activity** | |  | **Mediation effect of lipids on the physical activity-Insulin sensitivity association^3^** | | | |
| --- | --- | --- | --- | --- | --- | --- | --- | --- |
|  |  | **Beta (95%)¹** | **q value²** |  | **Total effect** | **Mediation effect** | **% mediated** | **p for % mediated** |
| AC(18:1) B | Acylcarnitine | **-0.044 (-0.076,-0.012)** | 0.05 |  | 0.097 (0.062,0.132) | -0.006 (-0.011,-0.002) | **-6.05 (-13.20,-1.92)** | **0.004** |
| CER(d34:0) | Ceramide | **-0.049 (-0.083,-0.016)** | 0.037 |  | 0.098 (0.063,0.132) | 0.009 ( 0.003, 0.015) | **8.96 (  3.60,16.40)** | **0.004** |
| CER(d34:1) | Ceramide | **-0.076 (-0.109,-0.042)** | 0.001 |  | 0.098 (0.063,0.132) | 0.010 ( 0.005, 0.016) | **9.93 (  5.39,17.55)** | **<0.001** |
| CER(d34:2) | Ceramide | **-0.063 (-0.097,-0.030)** | 0.008 |  | 0.097 (0.063,0.132) | -0.001 (-0.004, 0.001) | -1.20 ( -4.53, 0.99) | 0.236 |
| CER(d36:1) | Ceramide | **-0.079 (-0.113,-0.045)** | <0.001 |  | 0.098 (0.063,0.132) | 0.010 ( 0.005, 0.016) | **9.86 (  5.41,17.41)** | **<0.001** |
| CER(d42:2) A | Ceramide | **-0.056 (-0.090,-0.023)** | 0.022 |  | 0.097 (0.062,0.131) | 0.003 ( 0.000, 0.006) | **2.92 (  0.57, 7.06)** | **0.02** |
| FA(16:0) | Fatty acid | **-0.044 (-0.075,-0.013)** | 0.046 |  | 0.097 (0.064,0.131) | 0.000 (-0.002, 0.002) | 0.05 ( -1.95, 2.18) | 0.922 |
| LPC(20:1) | Lysophosphatidylcholine | **-0.050 (-0.083,-0.016)** | 0.037 |  | 0.097 (0.062,0.132) | -0.004 (-0.009,-0.001) | **-4.39 (-10.36,-0.64)** | **0.022** |
| LPC(22:6) | Lysophosphatidylcholine | **0.064 ( 0.033, 0.096)** | 0.003 |  | 0.097 (0.062,0.130) | 0.003 ( 0.001, 0.007) | **3.46 (  0.53, 8.22)** | **0.016** |
| PC(20:2/20:2) | Phosphatidylcholine | **0.049 ( 0.017, 0.080)** | 0.035 |  | 0.097 (0.063,0.130) | -0.001 (-0.004, 0.000) | -1.26 ( -4.59, 0.40) | 0.134 |
| PC(37:4) | Phosphatidylcholine | **0.053 ( 0.018, 0.088)** | 0.037 |  | 0.097 (0.062,0.131) | 0.007 ( 0.003, 0.012) | **7.39 (  2.84,14.86)** | **<0.001** |
| PC(38:3) | Phosphatidylcholine | **-0.051 (-0.084,-0.017)** | 0.037 |  | 0.097 (0.063,0.131) | 0.008 ( 0.003, 0.014) | **7.81 (  2.70,14.82)** | **0.004** |
| PC(38:6) A | Phosphatidylcholine | **0.056 ( 0.022, 0.091)** | 0.026 |  | 0.097 (0.063,0.131) | 0.002 ( 0.000, 0.005) | **2.02 (  0.04, 5.79)** | **0.042** |
| PC(40:5) B | Phosphatidylcholine | **-0.047 (-0.081,-0.013)** | 0.05 |  | 0.097 (0.062,0.131) | 0.005 ( 0.001, 0.009) | **4.53 (  1.22, 9.63)** | **0.012** |
| PC(p-16:0/22:6)/PC(o-16:1/22:6) | Ether-phosphatidylcholine | **0.077 ( 0.044, 0.110)** | <0.001 |  | 0.097 (0.063,0.130) | -0.002 (-0.005, 0.001) | -2.05 ( -6.26, 1.14) | 0.186 |
| PC(p-18:1/20:4)/PC(o-18:2/20:4) | Ether-phosphatidylcholine | **0.083 ( 0.049, 0.116)** | <0.001 |  | 0.097 (0.062,0.131) | 0.007 ( 0.003, 0.012) | **7.30 (  3.14,14.44)** | **<0.001** |
| PC(p-18:2/17:0)/PC(o-18:3/17:0) | Ether-phosphatidylcholine | **0.053 ( 0.020, 0.087)** | 0.027 |  | 0.097 (0.063,0.131) | 0.000 (-0.002, 0.002) | 0.09 ( -2.01, 2.54) | 0.918 |
| PC(p-22:3/18:5)/PC(o-22:4/18:5) | Ether-phosphatidylcholine | **0.054 ( 0.020, 0.088)** | 0.029 |  | 0.097 (0.062,0.131) | 0.004 ( 0.000, 0.008) | **3.96 (  0.39, 9.30)** | **0.028** |
| PC(p-34:2)/PC(o-34:3) | Ether-phosphatidylcholine | **0.058 ( 0.025, 0.092)** | 0.019 |  | 0.097 (0.063,0.131) | 0.003 ( 0.001, 0.007) | **3.35 (  0.82, 7.98)** | **0.004** |
| PC(p-36:4)/PC(o-36:5) | Ether-phosphatidylcholine | **0.054 ( 0.019, 0.090)** | 0.036 |  | 0.097 (0.063,0.131) | 0.002 ( 0.000, 0.005) | **2.08 (  0.07, 5.89)** | **0.04** |
| PC(p-36:5)/PC(o-36:6) | Ether-phosphatidylcholine | **0.056 ( 0.021, 0.090)** | 0.026 |  | 0.097 (0.063,0.130) | -0.001 (-0.003, 0.001) | -0.79 ( -3.68, 1.17) | 0.338 |
| PC(p-38:5)/PC(o-38:6) B | Ether-phosphatidylcholine | **0.055 ( 0.021, 0.089)** | 0.027 |  | 0.097 (0.062,0.131) | 0.004 ( 0.001, 0.008) | **3.93 (  0.75, 9.11)** | **0.018** |
| PE(18:0/22:5) A | Phosphatidylethanolamine | **-0.047 (-0.080,-0.014)** | 0.045 |  | 0.097 (0.062,0.131) | 0.008 ( 0.002, 0.014) | **7.80 (  2.75,14.80)** | **0.004** |
| PE(34:1) | Phosphatidylethanolamine | **-0.054 (-0.088,-0.020)** | 0.027 |  | 0.097 (0.063,0.131) | 0.010 ( 0.004, 0.017) | **10.12 (  4.54,18.24)** | **<0.001** |
| PE(36:1) A | Phosphatidylethanolamine | **-0.047 (-0.080,-0.014)** | 0.047 |  | 0.098 (0.062,0.131) | 0.011 ( 0.004, 0.019) | **11.21 (  4.19,20.18)** | **0.004** |
| SM(d39:1) B | Sphingomyelin | **0.074 ( 0.038, 0.111)** | 0.003 |  | 0.097 (0.063,0.131) | 0.001 (-0.001, 0.003) | 0.51 ( -1.20, 3.16) | 0.518 |
| SM(d44:2) A | Sphingomyelin | **-0.049 (-0.083,-0.015)** | 0.045 |  | 0.097 (0.062,0.132) | -0.001 (-0.004, 0.001) | -1.09 ( -4.22, 0.53) | 0.174 |
| TAG(50:2) | Triacylglycerol | **-0.052 (-0.087,-0.017)** | 0.037 |  | 0.096 (0.061,0.130) | 0.011 ( 0.003, 0.019) | **11.00 (  3.16,20.42)** | **0.012** |
| TAG(50:3) A | Triacylglycerol | **-0.048 (-0.082,-0.014)** | 0.045 |  | 0.097 (0.061,0.130) | 0.010 ( 0.002, 0.017) | **9.91 (  2.58,18.69)** | **0.012** |
| TAG(52:1) | Triacylglycerol | **-0.050 (-0.084,-0.015)** | 0.042 |  | 0.098 (0.062,0.132) | 0.015 ( 0.005, 0.024) | **14.91 (  5.66,26.03)** | **0.004** |
| TAG(53:3) | Triacylglycerol | **-0.048 (-0.082,-0.014)** | 0.048 |  | 0.097 (0.062,0.131) | 0.008 ( 0.002, 0.015) | **8.49 (  2.12,16.35)** | **0.014** |
| TAG(54:2) | Triacylglycerol | **-0.064 (-0.098,-0.030)** | 0.009 |  | 0.098 (0.062,0.132) | 0.016 ( 0.006, 0.026) | **16.15 (  6.88,27.40)** | **0.004** |
| TAG(54:3) | Triacylglycerol | **-0.067 (-0.101,-0.033)** | 0.005 |  | 0.097 (0.062,0.131) | 0.010 ( 0.004, 0.017) | **9.88 (  3.96,17.93)** | **0.004** |
| TAG(54:4) | Triacylglycerol | **-0.054 (-0.089,-0.020)** | 0.028 |  | 0.097 (0.062,0.131) | 0.007 ( 0.002, 0.013) | **6.88 (  1.95,13.78)** | **0.01** |
| TAG(55:4)/TAG(18:1/18:2/19:1) | Triacylglycerol | **-0.046 (-0.080,-0.013)** | 0.05 |  | 0.097 (0.062,0.131) | 0.007 ( 0.002, 0.013) | **7.20 (  2.24,14.13)** | **0.01** |
| TAG(56:3) | Triacylglycerol | **-0.057 (-0.091,-0.023)** | 0.022 |  | 0.098 (0.062,0.131) | 0.012 ( 0.005, 0.021) | **12.64 (  5.10,21.99)** | **0.004** |

The letter A, B or C at the end of lipid names indicates isomers. Boldface indicates statistical significance.

¹ Beta coefficient of lipids associated with habitual physical activity. Obtained by mixed-effects linear regression model, adjusting for age, sex, study center, education, BMI, fasting glucose, smoking, alcohol use, lipid-lowering medication use, and hypertension status at the study visit. Random effects were used to account for family relatedness and repeated measurements at baseline and follow-up visits.

² Adjusted for multiple testing using the Storey’s q-value method.

^3^ Mediation proportion of lipids was calculated as the average causal mediation effects relative to the total effect.

**Supplemental Table S4.** Plasma lipid species associated with physical activity levels (q<0.05) and diabetes risk in sensitivity analyses

| **Lipids** | **Class** | **Association with physical activity** | |  | **Associations with prediabetes or T2D, OR(95%)^3^** | | |
| --- | --- | --- | --- | --- | --- | --- | --- |
|  |  | **Beta (95%)^1^** | **q value²** |  | **T2D** | **Prediabetes** | **Prediabetes/T2D** |
| CE(18:1) | Cholesterol ester | 0.06 ( 0.02, 0.10) | 0.032 |  | **0.68 (0.46,1.00)** | **0.77 (0.62,0.96)** | **0.72 (0.59,0.88)** |
| **CER(d34:1)** | Ceramide | -0.07 (-0.10,-0.03) | 0.008 |  | **1.53 (1.16,2.02)** | 1.00 (0.83,1.21) | 1.16 (0.98,1.37) |
| **CER(d34:2)** | Ceramide | -0.06 (-0.10,-0.02) | 0.021 |  | 1.26 (0.95,1.67) | 0.84 (0.69,1.01) | 0.93 (0.78,1.10) |
| **CER(d36:1)** | Ceramide | -0.08 (-0.11,-0.04) | 0.003 |  | **1.48 (1.12,1.94)** | 1.10 (0.91,1.32) | **1.23 (1.04,1.45)** |
| **CER(d42:2) A** | Ceramide | -0.05 (-0.09,-0.02) | 0.037 |  | **1.36 (1.03,1.79)** | 1.03 (0.86,1.24) | 1.14 (0.96,1.34) |
| DAG(18:0/18:1) | Diacylglycerol | -0.05 (-0.08,-0.01) | 0.048 |  | **1.26 (1.02,1.56)** | 1.16 (0.99,1.36) | **1.24 (1.07,1.44)** |
| DAG(34:2) | Diacylglycerol | -0.05 (-0.08,-0.02) | 0.046 |  | **1.50 (1.20,1.87)** | 1.15 (0.97,1.36) | **1.32 (1.13,1.55)** |
| DAG(36:3) | Diacylglycerol | -0.05 (-0.08,-0.02) | 0.048 |  | **1.36 (1.07,1.72)** | 1.06 (0.88,1.27) | 1.17 (1.00,1.37) |
| GlcCer(d41:1) | Glucosylceramide | 0.05 ( 0.02, 0.09) | 0.044 |  | 1.15 (0.85,1.56) | 1.01 (0.84,1.23) | 1.05 (0.88,1.25) |
| **LPC(20:1)** | Lysophosphatidylcholine | -0.05 (-0.09,-0.02) | 0.038 |  | 1.15 (0.86,1.55) | 0.92 (0.75,1.13) | 0.99 (0.83,1.19) |
| **PC(20:2/20:2)** | Phosphatidylcholine | 0.05 ( 0.02, 0.08) | 0.042 |  | 1.04 (0.78,1.37) | 0.99 (0.82,1.20) | 1.01 (0.85,1.19) |
| **PC(38:6) A** | Phosphatidylcholine | 0.05 ( 0.02, 0.09) | 0.046 |  | 1.05 (0.76,1.45) | 0.94 (0.77,1.14) | 0.97 (0.81,1.16) |
| **PC(p-16:0/22:6)/PC(o-16:1/22:6)** | Ether-phosphatidylcholine | 0.09 ( 0.05, 0.12) | <0.001 |  | 1.01 (0.74,1.39) | 0.94 (0.77,1.14) | 0.95 (0.80,1.13) |
| **PC(p-18:1/20:4)/PC(o-18:2/20:4)** | Ether-phosphatidylcholine | 0.09 ( 0.06, 0.13) | <0.001 |  | 0.79 (0.57,1.08) | **0.81 (0.67,0.98)** | **0.78 (0.66,0.93)** |
| **PC(p-18:2/17:0)/PC(o-18:3/17:0)** | Ether-phosphatidylcholine | 0.05 ( 0.02, 0.09) | 0.037 |  | 0.94 (0.66,1.34) | 0.96 (0.78,1.18) | 0.95 (0.79,1.15) |
| **PC(p-22:3/18:5)/PC(o-22:4/18:5)** | Ether-phosphatidylcholine | 0.06 ( 0.03, 0.10) | 0.018 |  | 0.76 (0.54,1.07) | 0.88 (0.72,1.08) | **0.82 (0.69,0.98)** |
| **PC(p-34:2)/PC(o-34:3)** | Ether-phosphatidylcholine | 0.06 ( 0.02, 0.09) | 0.023 |  | 1.11 (0.81,1.51) | 0.94 (0.77,1.14) | 0.99 (0.83,1.18) |
| **PC(p-36:4)/PC(o-36:5)** | Ether-phosphatidylcholine | 0.07 ( 0.03, 0.10) | 0.018 |  | 1.03 (0.74,1.45) | 0.99 (0.81,1.21) | 0.99 (0.82,1.19) |
| **PC(p-36:5)/PC(o-36:6)** | Ether-phosphatidylcholine | 0.06 ( 0.02, 0.10) | 0.021 |  | 1.03 (0.75,1.43) | 1.01 (0.83,1.22) | 1.00 (0.84,1.19) |
| **PC(p-38:5)/PC(o-38:6) B** | Ether-phosphatidylcholine | 0.07 ( 0.03, 0.10) | 0.012 |  | 1.01 (0.74,1.39) | 0.93 (0.76,1.13) | 0.94 (0.79,1.12) |
| PE(18:0/20:3) | Phosphatidylethanolamine | -0.06 (-0.09,-0.02) | 0.032 |  | **1.57 (1.24,1.98)** | 1.14 (0.96,1.36) | **1.35 (1.15,1.60)** |
| **PE(18:0/22:5) A** | Phosphatidylethanolamine | -0.05 (-0.09,-0.02) | 0.034 |  | **1.45 (1.13,1.86)** | 1.05 (0.88,1.25) | **1.20 (1.02,1.40)** |
| **PE(34:1)** | Phosphatidylethanolamine | -0.05 (-0.09,-0.02) | 0.034 |  | **1.45 (1.13,1.86)** | 1.15 (0.97,1.38) | **1.31 (1.11,1.54)** |
| **PE(36:1) A** | Phosphatidylethanolamine | -0.05 (-0.09,-0.02) | 0.042 |  | **1.46 (1.17,1.82)** | **1.22 (1.04,1.43)** | **1.43 (1.22,1.68)** |
| **SM(d39:1) B** | Sphingomyelin | 0.08 ( 0.04, 0.12) | 0.004 |  | 1.06 (0.76,1.47) | 0.97 (0.80,1.19) | 0.99 (0.83,1.19) |
| **TAG(50:3) A** | Triacylglycerol | -0.05 (-0.09,-0.02) | 0.037 |  | **1.48 (1.13,1.93)** | 1.13 (0.94,1.35) | **1.26 (1.07,1.48)** |
| TAG(50:4) | Triacylglycerol | -0.05 (-0.09,-0.02) | 0.045 |  | **1.28 (1.02,1.63)** | 1.04 (0.87,1.25) | 1.14 (0.98,1.34) |
| TAG(51:4) | Triacylglycerol | -0.05 (-0.09,-0.02) | 0.046 |  | **1.31 (1.04,1.64)** | 1.02 (0.85,1.23) | 1.13 (0.97,1.33) |
| TAG(52:4)/TAG(16:1/18:1/18:2) | Triacylglycerol | -0.05 (-0.09,-0.02) | 0.042 |  | **1.29 (1.03,1.62)** | 0.97 (0.80,1.18) | 1.09 (0.93,1.28) |
| TAG(52:5) | Triacylglycerol | -0.05 (-0.09,-0.02) | 0.043 |  | **1.32 (1.03,1.68)** | 0.98 (0.81,1.18) | 1.09 (0.93,1.28) |
| **TAG(53:3)** | Triacylglycerol | -0.05 (-0.09,-0.02) | 0.046 |  | **1.34 (1.06,1.69)** | 1.12 (0.94,1.33) | **1.23 (1.05,1.44)** |
| **TAG(54:2)** | Triacylglycerol | -0.07 (-0.10,-0.03) | 0.011 |  | **1.39 (1.11,1.75)** | **1.21 (1.02,1.43)** | **1.35 (1.15,1.58)** |
| **TAG(54:3)** | Triacylglycerol | -0.07 (-0.10,-0.03) | 0.01 |  | 1.28 (0.97,1.69) | 1.11 (0.92,1.34) | **1.19 (1.00,1.40**) |
| **TAG(54:4)** | Triacylglycerol | -0.06 (-0.09,-0.02) | 0.032 |  | 1.15 (0.85,1.55) | 0.99 (0.82,1.21) | 1.04 (0.87,1.23) |
| **TAG(55:4)/TAG(18:1/18:2/19:1)** | Triacylglycerol | -0.05 (-0.09,-0.02) | 0.048 |  | **1.27 (1.04,1.54)** | 1.09 (0.92,1.28) | **1.19 (1.02,1.38)** |
| **TAG(56:3)** | Triacylglycerol | -0.06 (-0.10,-0.03) | 0.019 |  | **1.22 (1.00,1.48)** | 1.09 (0.93,1.28) | **1.19 (1.02,1.38)** |

Lipids in boldface are significant (q < 0.05) in both main analyses and sensitivity analyses. Associations in boldface indicate statistical significance. The letter A, B or C at the end of lipid names indicates isomers.

¹ Beta coefficient of lipids associated with habitual physical activity. Obtained by mixed-effects linear regression model, adjusting for age, sex, study center, education, BMI, fasting glucose, smoking, alcohol use, lipid-lowering medication use, hypertension status at the study visit and diet quality assessed by the Alternative Healthy Eating Index 2010 (AHEI). Random effects were used to account for family relatedness and repeated measurements at baseline and follow-up visits.

² Adjusted for multiple testing using the Storey’s q-value method.

^3^ Odds ratio of baseline lipids associated with incident outcomes. Obtained by mixed-effects logistic models, adjusting for all covariates above (baseline level).

**Supplemental Figure S1.** Participant selection and analyses flowchart

**
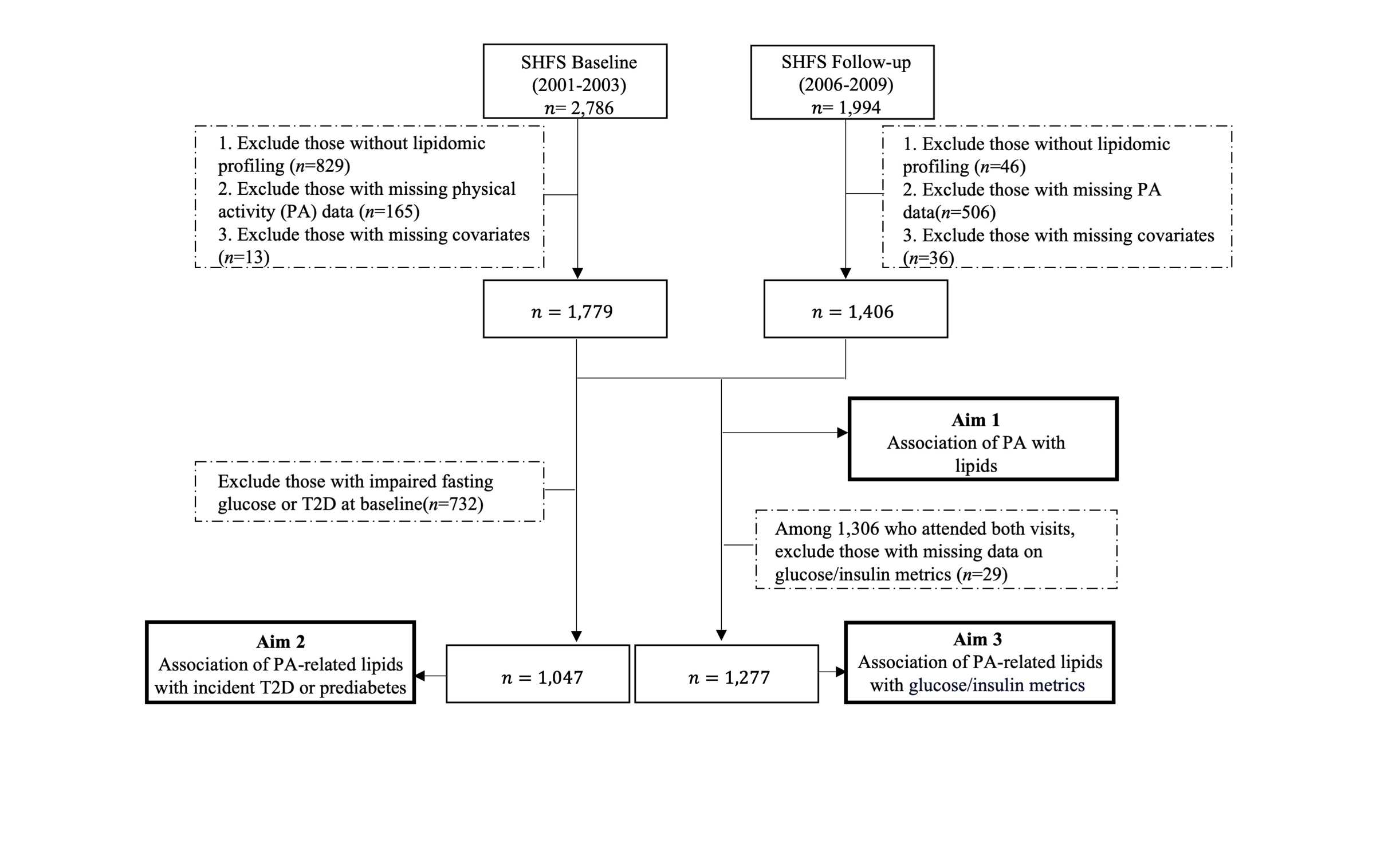
**

**Supplemental Figure S2.** Plasma lipid species associated with physical activity levels (q<0.05), incident prediabetes and combined incidence of T2D/prediabetes


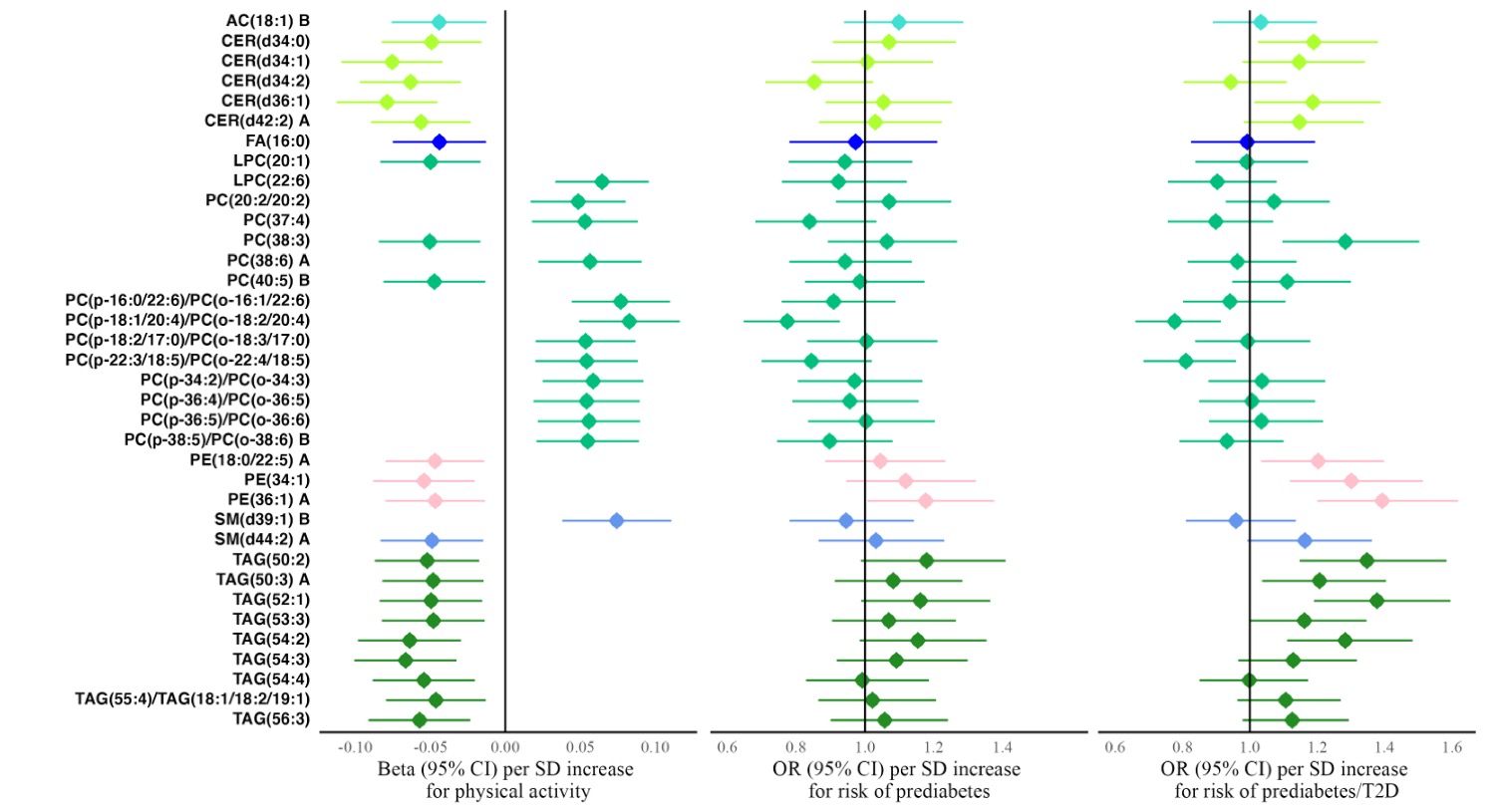


Beta coefficients of lipids associated with physical activity were obtained by mixed-effects linear regression models, adjusting for age, sex, study center, education, BMI, fasting glucose, smoking, alcohol use, lipid-lowering medication use, and hypertension status at the study visit. Odds ratios of baseline lipids associated with incident prediabetes or combined incidence of prediabetes/T2D were obtained by mixed-effects logistic models, adjusting for all covariates above (baseline level). The letter A or B at the end of lipid names indicates isomers. Abbreviations: AC, acylcarnitine; CER, Ceramide; FA, fatty acid; LPC, lysophosphatidylcholine; OR, odds ratio; PC, phosphatidylcholine; PE, phosphatidylethanolamine; SD, standard deviation; SM, sphingomyelin; TAG, triacylglycerol.

**Supplemental Figure S3.** Pathway enrichment results for physical activity-related lipids (P<0.05)

**
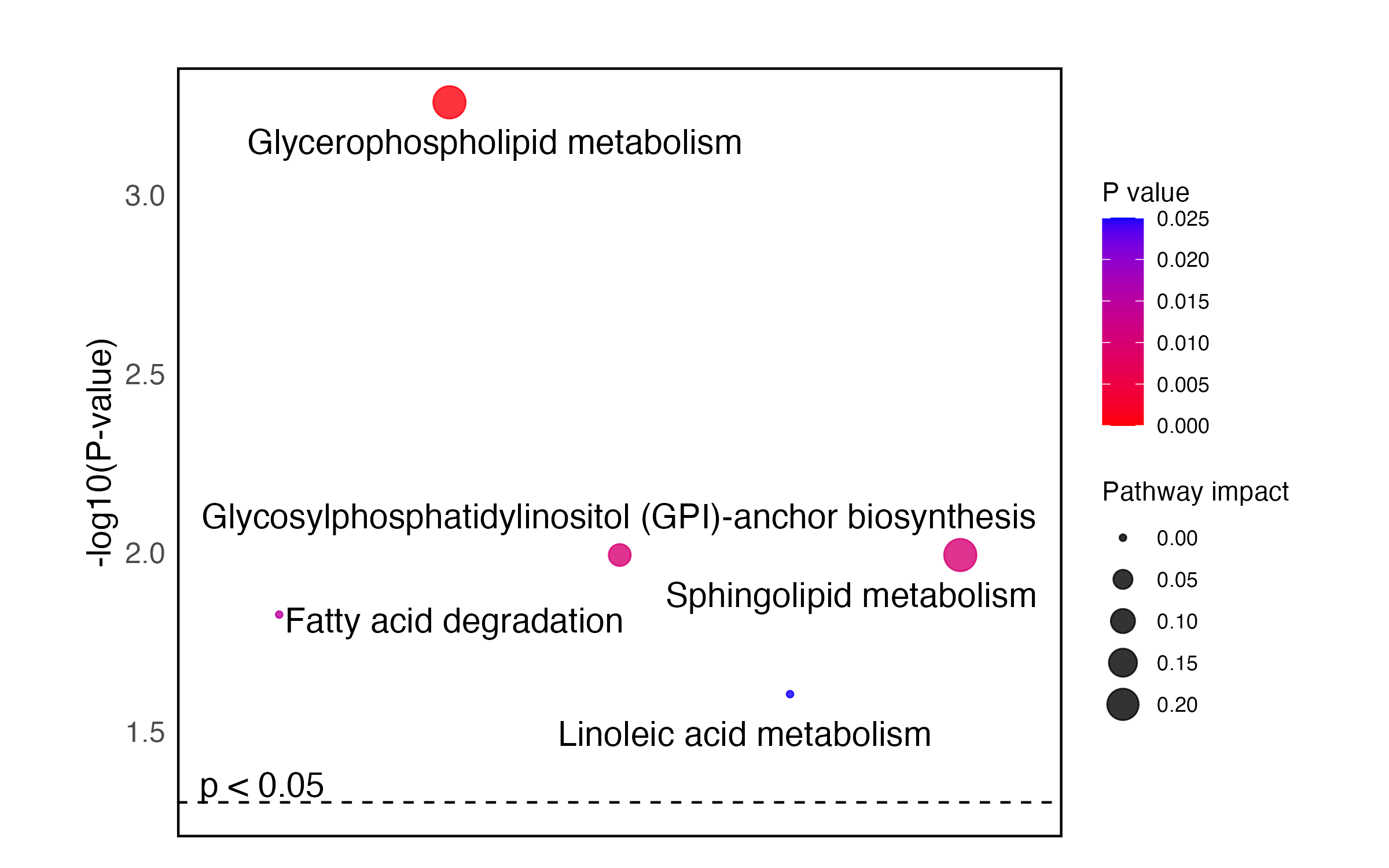
**

X-axis: Metabolic pathways; Y-axis: -log10^P^. Analyses were conducted using MetaboAnalyst 6.0 platform. The statistical significance of each pathway was evaluated using the Fisher’s exact test. The importance of each pathway was measured by topological analysis. The pathway impact score was calculated by dividing the sum of the importance measures of the identified lipids by the total importance measures of all metabolites within the pathway. All pathways with P < .05 are shown; glycerophospholipid metabolism remained significant after false discovery rate correction (FDR <.05) .

**Supplemental Figure S4.** Discriminative ability of the lipidomic score

**(A)** Violin plot of lipidomic score by physical activity status. Median values are displayed for each group. P value for group difference was obtained by one-way ANOVA. Physical activity status of poor, intermediate and ideal was defined as <3,500, 3,500-10,000 and >10,000 steps/day, respectively.

**(B)** ROC curve for lipidomic score classifying poor vs ideal physical activity. Area under the ROC curve (AUC) value and 95% CI are displayed on the curve.


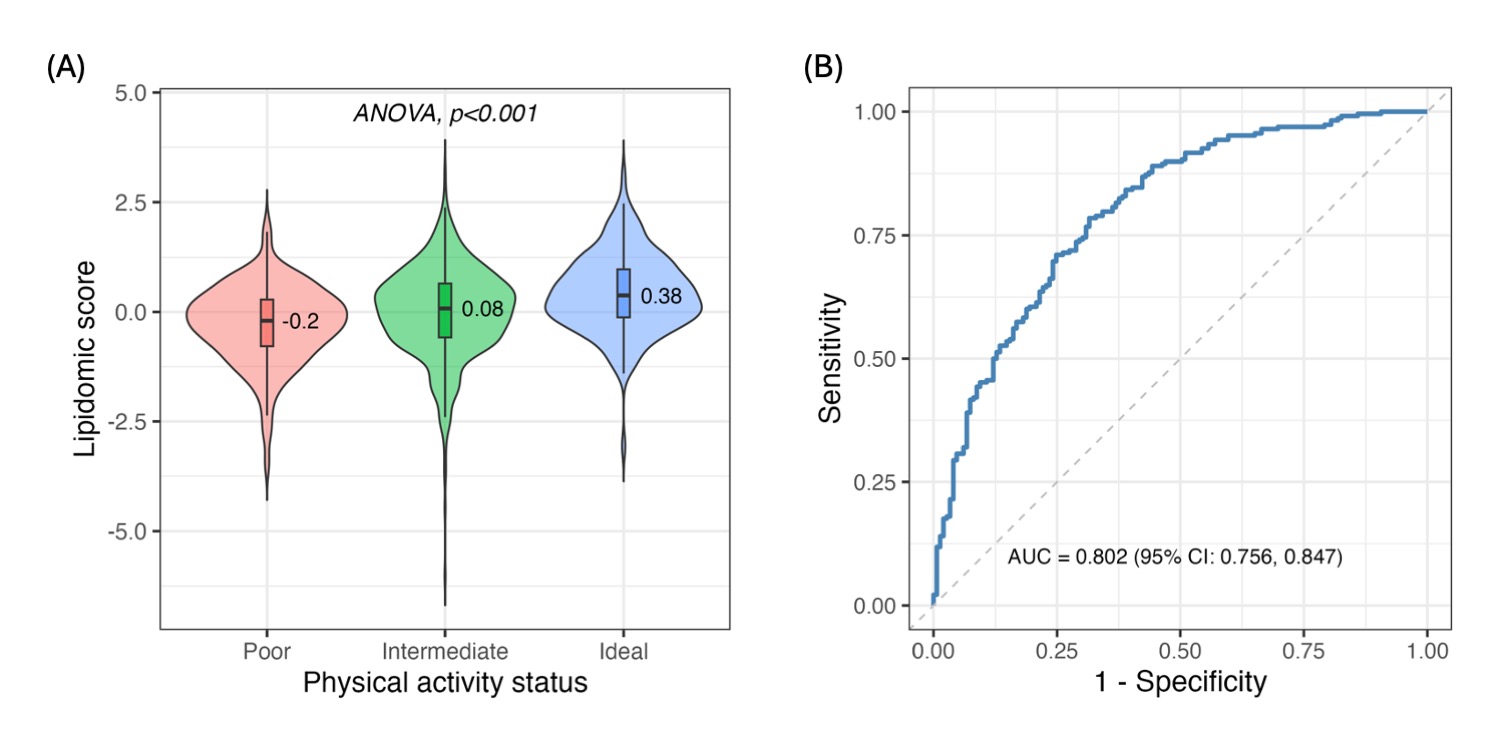


**Supplemental Figure S5.** Baseline lipidomic score is associated with diabetes risk

**(A)** Associations between lipidomic score and incident diabetes or prediabetes.

**(B)** Associations between lipidomic score and glucose/insulin metrics.


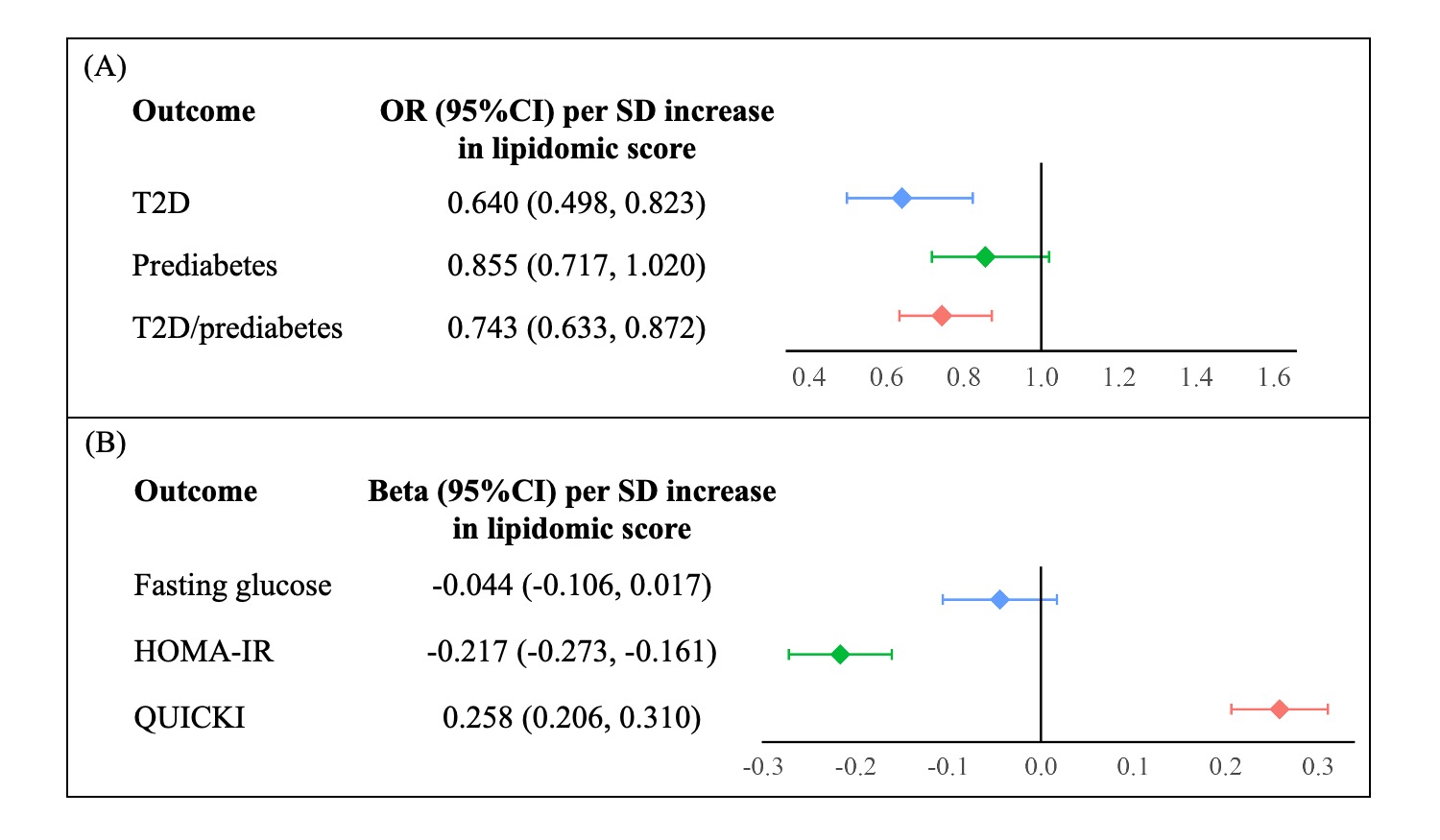


The lipidomic score was calculated as the sum of standardized lipid concentrations weighted by their corresponding regression coefficients from single lipid models. The significant lipids (known) at q<0.05 were used. Odds ratios were obtained by mixed-effects logistic regression models, and regression coefficients were obtained from mixed-effects linear regression models, adjusting for the same covariates in the single lipid models (baseline level). Abbreviations: OR, odds ratio; SD, standard deviation.
